# Virtual screening of potentially endocrine-disrupting chemicals against nuclear receptors and its application to identify PPARγ-bound fatty acids

**DOI:** 10.1007/s00204-020-02897-x

**Published:** 2020-09-09

**Authors:** Chaitanya K. Jaladanki, Yang He, Li Na Zhao, Sebastian Maurer-Stroh, Lit-Hsin Loo, Haiwei Song, Hao Fan

**Affiliations:** 1 Bioinformatics Institute (BII), Agency for Science, Technology, and Research (A*STAR), 30 Biopolis Street, Matrix No. 07-01, Singapore, 138671 Singapore; 2 Toxicity Mode-of-Action Discovery (ToxMAD) Platform, Innovations in Food and Chemical Safety Programme, Agency for Science, Technology, and Research (A*STAR), Singapore, 138671 Singapore; 3 Institute of Molecular and Cell Biology, 61 Biopolis Drive, Singapore, 138673 Singapore

**Keywords:** Virtual screening, In silico toxicity prediction, Nuclear receptors, EDC, ToxCast, Furan fatty acid

## Abstract

Nuclear receptors (NRs) are key regulators of energy homeostasis, body development, and sexual reproduction. Xenobiotics binding to NRs may disrupt natural hormonal systems and induce undesired adverse effects in the body. However, many chemicals of concerns have limited or no experimental data on their potential or lack-of-potential endocrine-disrupting effects. Here, we propose a virtual screening method based on molecular docking for predicting potential endocrine-disrupting chemicals (EDCs) that bind to NRs. For 12 NRs, we systematically analyzed how multiple crystal structures can be used to distinguish actives and inactives found in previous high-throughput experiments. Our method is based on (i) consensus docking scores from multiple structures at a single functional state (agonist-bound or antagonist-bound), (ii) multiple functional states (agonist-bound and antagonist-bound), and (iii) multiple pockets (orthosteric site and alternative sites) of these NRs. We found that the consensus enrichment from multiple structures is better than or comparable to the best enrichment from a single structure. The discriminating power of this consensus strategy was further enhanced by a chemical similarity-weighted scoring scheme, yielding better or comparable enrichment for all studied NRs. Applying this optimized method, we screened 252 fatty acids against peroxisome proliferator-activated receptor gamma (PPARγ) and successfully identified 3 previously unknown fatty acids with Kd = 100–250 μM including two furan fatty acids: furannonanoic acid (FNA) and furanundecanoic acid (FUA), and one cyclopropane fatty acid: phytomonic acid (PTA). These results suggested that the proposed method can be used to rapidly screen and prioritize potential EDCs for further experimental evaluations.

**Electronic supplementary material:**

The online version of this article (10.1007/s00204-020-02897-x) contains supplementary material, which is available to authorized users.

## Introduction

Endocrine-disrupting chemicals (EDCs) are chemicals that can interfere with the natural hormonal systems in the body via various mechanisms, including altering the production, release, transport, binding, and metabolism of key hormones responsible for energy homeostasis, body development, and sexual reproduction (Weatherman et al. 1999; Sanderson 2006; Bain et al. 2007; Reif et al. 2010; Huang et al. 2010; Soto and Sonnenschein 2010; Rotroff et al. 2013; Toporova and Balaguer 2020). Humans are exposed to many potential EDCs that can be found in the environment, food, and consumer products. Therefore, it is not surprising that the identification of potential EDCs has been a central focus in predictive toxicology. The European Commission in Regulations, Registration, Evaluation, Authorization, and Restriction of Chemical (REACH) (European Council 2006) has listed EDCs as important toxicological endpoints for chemical registrations (Nicolotti et al. 2014). Moreover, the United States Environmental Protection Agency (US-EPA) is also focusing on EDCs under the Endocrine Disruptor Screening Program (EDSP).

EDCs may disrupt normal functions of the endocrine system by interacting with nuclear receptors (NRs) (Diamanti-Kandarakis et al. 2009; Reif et al. 2010; Soto and Sonnenschein 2010; Schug et al. 2011; Rotroff et al. 2013). For example, bisphenol-A (BPA) and its analogs, heavily used in the manufacture of polycarbonate plastics and epoxy resins, have been shown to bind to estrogen (ER) and androgen receptors (AR). The NR superfamily are ligand-activated transcription factors that regulate various physiological processes such as cell development, differentiation, proliferation, and metabolism. They are also associated with numerous pathologies such as reproductive abnormalities, inflammation, cardiovascular disease, and cancer. (Ribeiro et al. 1995; Bain et al. 2007). NRs are known to be activated by hormones, vitamins, fatty acids, and metabolites in the body. Members of this superfamily contain a N-terminal transactivation domain (NTD), a zinc-finger DNA binding domain (DBD), and a C-terminal ligand-binding domain (LBD). The binding of ligands with their associated NR transactive specific genes within a target tissue Ligand binding to its correlated NR results in the transactivation of specific genes within a target tissue (Weatherman et al. 1999; Bain et al. 2007). NRs may be subdivided into three mechanistic classes. Class I NRs, also called steroid receptors, include the ER, AR, progesterone receptor (PR), mineralocorticoid receptor (MR), and glucocorticoid receptor (GR). Class II NRs include the thyroid hormone receptors (TRα and β), peroxisome proliferator-activated receptors (PPARα, β, and γ), retinoic acid receptors (RARα, β, and γ), liver X receptors (LXRα and β), vitamin D receptor (VDR), and RAR-related orphan receptors (RORα, β, and γ) (Robinson-Rechavi et al. 2003; Bain et al. 2007). The members of this subfamily heterodimerize with retinoid X receptors (RXRα, β, and γ). Class III NRs are a family of the orphan receptors. This NR class includes a group of proteins that share substantial sequence homology with known NRs but have not yet identified the ligands, such as small heterodimer partner (SHP), testicular receptor 2 and 4 (TR2 and 4), and estrogen-related receptor (ERRα, β, and γ).

Despite the known importance of NRs and their natural ligands in regulating endocrine systems, the relatively large number of proteins in this family (many of which are still poorly studied) and the huge numbers of potential EDCs with high human exposure levels have made the efforts to identify NR-bound EDCs to be very challenging. Traditional animal studies are expensive, time consuming, and low throughput (European Council 2006; Dix et al. 2007; Judson et al. 2008, 2009; Cohen Hubal et al. 2010; Knudsen et al. 2011; Kavlock et al. 2012). Thus, most previous work has been focused on a few well-characterized NRs, such as ER, AR, and PR, and a small number of chemicals of concerned, such as BPA. To allow the screening of the biological activity of large numbers of chemicals more effective, US-EPA and several other agencies had initiated the Toxicity Forecaster (ToxCast) program and the Tox21 consortium. Thousands of chemicals were screened and analyzed using high-throughput in vitro biochemical and cell-based assays that cover many key cellular pathways and biochemical targets relevant to toxicology (Dix et al. 2007). Several highly predictive in vitro cell-type-specific toxicity models based on phenotypic profiling have also been developed by the Agency for Science, Technology, and Research (A*STAR) from Singapore (Su et al. 2016; Lee et al. 2018; Paul Friedman et al. 2020; van der Ven et al. 2020; Hussain et al. 2020). These in vitro screening efforts have generated a large amount of invaluable bioactivity data for the tested chemicals, but many relevant environmental agents and/or food components are still not being tested. Therefore, computational or in silico methods have the potential to bridge the gap and predict the bioactivities of relevant chemicals with little or without any experimental data.

Most existing computational methods for predicting potential EDCs are based on quantitative structure–activity relationship (QSAR) models (Shi et al. 2001; Nicolotti et al. 2008, 2009; Capuzzi et al. 2016) that require high chemical similarity to known binders (Capuzzi et al. 2016). However, the accuracy of these methods can be limited when applied to chemicals that belong to different scaffolds from known EDCs. To overcome this issue, protein structure-based docking or virtual screening can be employed, benefiting from the availability of three-dimensional structures of target receptors and providing insights into molecular recognition events. Such methods, primarily used in pharmaceutical ligand discovery programs, are intended to search large libraries of small molecules to suggest possible chemicals that can bind to a protein target with high affinity (Kitchen et al. 2004; Shoichet 2004; Nicolotti et al. 2008). To date, these approaches have not been widely used in toxicology, especially for the screening of potential EDCs from large numbers of chemicals. Given the enormous and fast*-*growing mass of protein structures and in vitro bioactivity data determined experimentally, structure-based docking or virtual screening method might provide an excellent tool to rapidly flag out potential EDCs for further experimental evaluations or confirmations of their adverse effects.

We have developed a structure-based virtual screening method to identify active EDCs against twelve human NRs (Weatherman et al. 1999; Bain et al. 2007; Huang et al. 2010; Toporova and Balaguer 2020) with ToxCast NR activity data, as well as multiple (≥ 2) agonist-bound and antagonist-bound crystal structures of receptors. These NRs include AR, GR, PR, ERα, ERβ from the steroid receptor class, and PPARα, PPARγ, RARα, RORγ, VDR, RXRα, LXRβ from the thyroid/retinoid receptor class. Here, we present the method and its benchmark results using the ToxCast NR activity data, and its application to identify PPARγ-bound fatty acids. Only a few previous studies had used molecular docking or virtual screening to predict the potential toxicological effects of NR ligands (Trisciuzzi et al. 2015, 2017). For example, Trisciuzzi et al. demonstrated that structure-based approaches, such as molecular docking, could be extended to exploratory toxicology studies, using ToxCast estrogenic potential and androgenic potential data (Trisciuzzi et al. 2015, 2017). Due to the aforementioned limitations, these studies focused only on one NR and failed to provide a complete picture of the capability of structure-based methods for EDC prediction. Furthermore, the protein target’s flexibility is typically not taken completely into consideration (Knegtel et al. 1997; Fradera et al. 2002; Carlson 2002; McCammon 2005; Cavasotto and Singh 2008; Cozzini et al. 2008; Ma et al. 2009; Spyrakis and Cavasotto 2015). In our current study, for each of the 12 selected receptors, we included 2 agonist-bound and 2 antagonist-bound crystal structures, to systematically evaluate the utility of multiple receptor structures in EDC prediction using virtual screening.

To gain further insights on the use of virtual screening for EDC prediction, we explored the following questions. First, since multiple ligands are known for each receptor and ligand-based methods are orthogonal to the protein-based methods, we want to know whether the proposed method can better discriminate ToxCast actives from inactives when docking scores of screened chemicals are weighted by their chemical similarities to the known ligands. Second, given the fact that alternative binding pockets (ABP) have been confirmed for nuclear receptors such as AR and RORγ, we investigated the possible impact of virtual screening against ABPs on ToxCast active prediction accuracy. Lastly, we performed a case study on fatty acids to demonstrate how the proposed method can be used in practice. Fatty acids are a large group of food components that can be found naturally in seafood, nuts and seeds, and plant oils, but also increasingly be used as food additives. They play important roles in human health and nutrition and some of them have been found active against nuclear receptors such as PPARγ (Kliewer et al. 1997; Xu et al. 1999; Kersten et al. 2000; Sampath and Ntambi 2004; Manco et al. 2004; Bordoni et al. 2006; Madrazo and Kelly 2008; Marion-Letellier et al. 2016). However, only 14 of them have been experimentally tested in the ToxCast program. We used the proposed method to screen 252 dietary-oriented fatty acids against PPARγ and experimentally verified that three of the top novel hits bind to PPARγ using surface plasmon resonance analysis. Together, our results demonstrate the feasibility of using virtual screening to prioritize suspected EDCs for further experimental evaluations.

## Materials and methods

### ToxCast chemical benchmarking database

The benchmarking database for all the NRs was obtained from the ToxCast database consisting of a curated repository of chemicals with high-quality experimental data (https://www.epa.gov/chemical-research/toxicity-forecaster-toxcasttm-data). The Tox21/ToxCast database released in October 2015, containing information for 9076 chemicals tested across 1193 different assays, including chemical names, CAS numbers, 2D structures, quality control grades, descriptions of the assays, and results summarized by AC50 values. These assays have been developed across multiple human and animal cell lines by several providers, including Attagene Inc. (ATG, one transactivation assay measuring reporter RNA transcript levels), NIH Chemical Genomics Center (Tox21, three transactivation assays measuring reporter protein level readouts), and NovaScreen (NVS, biochemical radioligand binding assays), BioSeek (BSK). In all assays, for each chemical–assay combination, a micromolar concentration was reported as the negative logarithm of the half-maximal activity concentration (pAC50), chemicals with “1” values for a given assay considered as active and with “0” values considered as inactive. For AR, PR, GR, PPARα, PPARγ, RARα, we selected chemicals that were screened in NVS assays because these chemicals were tested by biochemical radioligand binding assays that provide a measure of interaction between protein and ligands, as well as the degree of affinity (weak, strong, or no binding). For other nuclear receptors that do not have biochemical data we selected chemicals screened by cell-based assays, for RORγ, VDR, RXRα, and LXRβ from ATG and for ERα and ERβ from Tox21 (Table 1). Furthermore, we discarded chemicals without defined structures (e.g. mixtures, oils), chemicals without a molecular description of their structure in SMILES (Simplified molecular-input line-entry system), and chemicals with less than 5 non-hydrogen atoms.

**Table 1:** Nuclear receptor targets

Protein	Active structure PDB ID	Inactive structure PDB ID	Actives	Inactives	Assay
AR*	3L3X; 2AX9	3RLJ; 2OZ7	97	2561	Biochemical assay, single-readout assay that uses extracted gene-proteins from MCF7 in a cell-free assay. Measurements 18 h after chemical dosing in a 96-well plate
GR	3K22; 6EL9	1NHZ; 4MDD	207	2351	Biochemical assay, single-readout assay that uses extracted gene-proteins in a cell-free assay. Measurements were taken 16 h after chemical dosing in a 96-well plate
PR	1SQN; 3KBA	2OVH; 4OAR	57	2403	Biochemical assay, single-readout assay that uses extracted gene-proteins from T47D in a cell-free assay. Measurements were taken 18 h after chemical dosing in a 96-well plate
ERα	1X7R; 5U2D	1XP1; 2IOK	119	1651	Biochemical assay, single-readout assay that uses extracted gene-proteins from MCF7 in a cell-free assay. Measurements 18 h after chemical dosing in a 96-well plate
ERβ	3OLL;1U3R	1L2J; 1NDE	198	1582	Biochemical assay, single-readout assay that uses extracted gene-proteins from MCF7 in a cell-free assay. Measurements were taken 18 h after chemical dosing in a 96-well plate
PPARα	2P54; 1I7G	1KKQ; 2REW	68	1701	Biochemical assay, single-readout assay that uses extracted gene-proteins in a cell-free assay. After 1 h after chemical dosing in a 384-well plate
PPARγ	3BC5; 3VSO	5DWL; 5LSG	108	1660	Biochemical assay, single-readout assay that uses extracted gene-proteins in a cell-free assay. Measurements were taken 2 h after chemical dosing in a 384-well plate
RARα	3KMR; 3A9E	1DKF; 5K13	48	1717	Biochemical assay, single-readout assay that uses extracted gene-proteins in a cell-free assay. Measurements were taken 2 h after chemical dosing in a 384-well plate
RORγ^#^	3LOL; 4WLB	5NTK; 5K3N	57	3265	Cell-based assay, multiplexed-readout assay that uses HepG2, a human liver cell line, with measurements taken at 24 h after chemical dosing in a 24-well plate
VDR	3B0T:3AZ2	5XPL:5XUQ	951	2377	Cell-based assay, multiplexed-readout assay that uses HepG2, a human liver cell line, with measurements taken at 24 h after chemical dosing in a 24-well plate
RXRα	1MVC; 5LYQ	3NSQ; 2P1V	124	3204	Cell-based assay, multiplexed-readout assay that uses HepG2, a human liver cell line, with measurements taken at 24 h after chemical dosing in a 24-well plate
LXRβ	1P8D; 3KFC	6S4U; 6S4N	102	3226	Cell-based assay, multiplexed-readout assay that uses HepG2, a human liver cell line, with measurements taken at 24 h after chemical dosing in a 24-well plate

*AR* androgen receptor, *GR* glucocorticoid receptor, *PR* progesterone receptor, ERα estrogen receptor alpha, *ERβ* estrogen receptor beta, *PPARα* peroxisome proliferator-activated receptor alpha, *PPARγ* peroxisome proliferator-activated receptor gamma, *RARα* retinoic acid receptor alpha, *RORγ* retinoid-related orphan receptor-gamma, *VDR* vitamin D receptor, *RXRα* retinoic X -receptor alpha

*For AR, PDB ID 2PIW was used for docking at alternative binding pocket 1 (ABP1), for other proteins another alternative pocket was extrapolated based on AR allosteric site

^#^For RORγ, PDB ID 5C4T was used for docking at alternative binding pocket 2 (ABP2), for other proteins an alternative pocket was extrapolated based on RORγ allosteric site

### Protein structure database

The X-ray structures of the 12 NRs used in this benchmarking study were retrieved from the RCSB PDB database (Table 1) (https://www.rcsb.org/). For each NR, two agonist-bound structures and two antagonist-bound structures with high resolution were selected. In addition, we included one more AR structure (PDB ID: 2PIW) and one more RORγ structure (PDB ID: 5C4T), each containing a ligand bound to a unique alternative binding pocket (ABP1 and ABP2, respectively). The best resolution crystal structure was used for the remaining NRs to extrapolate the AR and RORγ alternative binding sites.

### Fatty acid prediction database

The fatty acid (FA) library is curated from various sources such as lipidbank (https://lipidbank.jp/), seed oil fatty acid database (SOFA), (Matthäus 2012) and lipidHome (Foster et al. 2013). The fatty acids can be categorized into 10 groups, based on their chemical structures, including saturated, unsaturated, branched, hydroxy, keto, thia, epoxy, cyclopropane, acetylenic and furanoid fatty acids. Their chain lengths range from medium (C6–C12), long (C13–C21), to very long (C22 and above). The majority of fatty acids occur naturally in the diet and in the body. For each of the 252 fatty acids, the name, the number of carbon atoms, and dietary source are summarized in Table S1.

### Molecular docking

All the protein structures were preprocessed by the protein preparation wizard module (Protein Preparation Wizard, Schrödinger, LLC, NewYork, NY). The 3D structures for all the ToxCast chemicals and fatty acids were prepared using LigPrep (LigPrep, Schrödinger, LLC, New York, NY). Then molecular docking was performed using the Glide module (Glide, Schrödinger, LLC, New York, NY). Firstly, to set up receptor grid, the Receptor Grid Generation Panel within the Glide suite was used to define one cubic grid box (15 Å per side) as the inner-box and another cubic box (20 Å per side) as the outer-box, both centering at the centroid of the crystal ligand. The OPLS3e force field was employed for identifying and ranking the docking poses.

### Chemical similarity-weighted scoring scheme

**Fig. 1:**
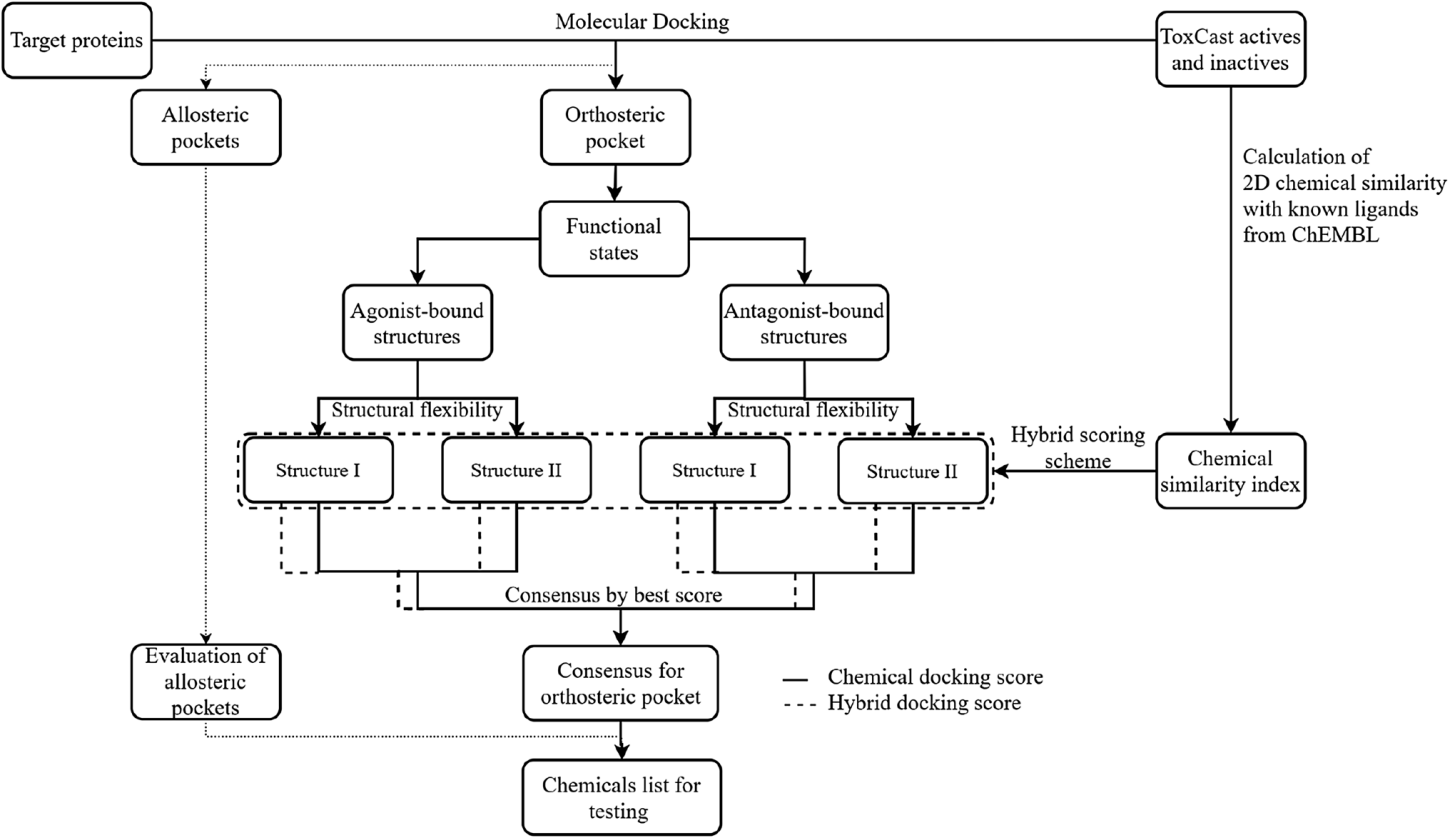
Work-flow of in silico toxicity prediction targeting nuclear receptors

Known ligands of the 12 NRs were extracted from ChEMBL (https://www.ebi.ac.uk/chembl/), using a filter that binding affinity value (Ki, Kd, EC50 and IC50) was less than 100 μM. The linear fingerprints (daylight method) of the ToxCast chemicals and extracted ChEMBL ligands of the 12 NRs were generated and compared using canvas module (Canvas, Schrödinger, LLC, New York, NY) yielding the Tanimoto coefficient (Tc) metrics. For each ToxCast chemical included for one specific NR, multiple Tc values were generated with respect to various ligands of that NR, and the highest Tc value was used for that chemical. The chemical similarity-weighted scoring scheme was constructed by applying conditional weighting factor to the docking score of a chemical based on its Tc value, as stated in Eq. 1 (Fig. 1).

1 $${\text{E}}_{{{\text{DOCK}}}}^{\prime } = \left( {1 + {\text{i}}} \right){\text{*E}}_{{{\text{DOCK}}}} \quad \left\{ {\begin{array}{*{20}l} {i = 0.25 \quad if \, Tc > 0.7} \\ {i = 0.10 \quad if \, 0.3 \le Tc \le 0.7} \\ {i = 0 \quad if \, Tc \, < 0.3} \\ \end{array} } \right..$$

### Evaluation of virtual screening results

The accuracy of the virtual screening was assessed using enrichment factor (EF) and logarithmic area under curve (logAUC), as described in Eqs. 2 and 3 (Fan et al. 2009, 2012; Mysinger and Shoichet 2010; Mysinger et al. 2012; Lim et al. 2018). The EF is the concentration of the true positives among the top-scoring docking hits compared to their concentration throughout the entire database:

2 $${\text{EFsubset}} = \frac{{\left( {{\text{Active}}_{{{\text{selected}}}} /N_{{{\text{subset}}}} } \right)}}{{\left( {{\text{Active}}_{{{\text{total}}}} /N_{{{\text{total}}}} } \right)}}.$$

In this study, EF_1_ (enrichment factor at 1% of the ranked database) was measured. To quantify the enrichment independently of the arbitrary value of *N*_subset_, we also calculated the area under the enrichment curve with *x*-axis on a logarithmic scale to favor early enrichment (logAUC):

3 $${\text{logAUC}} = \frac{1}{{{\log}_{10} 100/0.1}}\mathop \sum \limits_{0.1}^{100} \frac{{{\text{Active}}_{{\text{selected }}} \left( x \right) }}{{{\text{Active}}_{{{\text{total}}}} }} \Delta x \, and \, x = {\log}_{10} \frac{{N_{{{\text{subset}}}} }}{{N_{{{\text{total}}}} }},$$

where ∆*x* is 0.1. A random selection of true positives from the database will yield a logAUC value of 14.5 while a mediocre selection picking twice as many ToxCast actives than random yields a logAUC value of 24.5. Both EF_1_ and logAUC were considered significantly different, when one value is over 10% larger (better) or smaller (worse) than the other, otherwise these two values are considered to be comparable.

For each NR, both EF_1_ and logAUC were computed for each receptor structure, as well as consensus over multiple receptor structures. The consensus score was calculated by ranking each compound in the database using its best energy across all receptor structures (Fan et al. 2009; Lim et al. 2018).

### Experimental assays

#### Protein expression and purification

PPARγ ligand binding domain (204–477) was cloned into pGEX-6p-1 vector (GE Healthcare) and expressed as GST fusion protein in E. Coli-BL21(DE3) strain (Agilent Technologies). The protein was firstly purified using a glutathione Sephararose 4B column, followed by PreScission protease cleavage to remove the GST-tag. The cleaved fusion protein was further purified to homogeneity by desalting, glutathione Sephararose 4B and Supperdex 75 (GE Healthcare) columns. The finally collected protein in 20 mM Tirs, pH8.0, 150 mM NaCl and 2 mM DTT was concentrated to 20 mg/ml. The purity was checked by SDS-PAGE and protein was stored at − 80 °C for use.

#### Surface plasmon resonance (SPR) analysis

PPARγ (0.05 mg/ml) were coupled onto CM5 sensor chips according to amine coupling procedure from the manufacturer’s manual. The final immobilization level of PPARγ was about 6500 resonance units (RUs). A reference channel was generated at same conditions without protein injection and used as a blank control to correct the instrument or buffer artifacts. Fatty acids were dissolved in DMSO and diluted with concentration ranging from 0.78 to 50 μM, and injected at a flowrate of 40 μL/min. Each sensorgram consists of an association phase (120 s), indicating the binding of the injected compound to the protein, followed by a dissociation phase (300 s) during which the running buffer (10 mM phosphate buffer, 150 mM NaCl, 5% DMSO, 0.05% Tween 20, pH 7.4) is passed over and the bound compounds were eluted from the chip surface. The Kd was calculated by steady-state binding fitting method in Biacore T200 evaluate the software.

## Results and discussion

### Cognate ligand docking

To evaluate the accuracy of the docking method, we first docked the cognate ligands (ligands that bind to proteins in crystal structures) back into respective crystal structures of each target. Root mean square deviation (RMSD) was calculated between the ligand docking pose and its crystal structure. The RMSD values are shown in Table S2. We found that the current docking method reproduced the ligand crystal structures accurately (RMSD < 2.0 Å in 49 out of 50 structures, RMSD < 2.5 Å in all 50 structures).

### Virtual screening and ToxCast active enrichment

NRs are a family of ligand-regulated transcription factors, whose activities are mediated by a number of extracellular lipophilic ligands, including many key steroid hormones and metabolites in the endocrine systems. These receptors also exist in two distinct functional states: agonist-bound (active) state and antagonist-bound (inactive) state. We docked ToxCast actives and inactives to both functional states of the 12 NRs in this study, considering two NR structures for each functional state (in total 48 structures, Table 1)*.* First, we assessed the ToxCast active enrichment measured by EF1 and logAUC, from docking against a single receptor structure (Table 2). All the docking screens outperformed the random selection, with 55% structures outperformed the mediocre selection (twice better than random). For example, two known EDCs, Bisphenol A (BPA) and Diallyl phthalate (DAP), are ranked 63 and 254, respectively, out of 1770 chemicals against ER; and are ranked 173 and 128 out of 1768 chemicals against PPARγ in best-performing structures, respectively.

**Table 2:** ToxCast active enrichment from every single structure, and the consensus over multiple structures

Receptor	Active structures			Inactive structures			Total consensus logAUC (EF1)
	PDB ID	logAUC (EF1)	Concensus logAUC (EF1)	PDB ID	logAUC (EF1)	Consensus logAUC (EF1)	
AR	3L3X	35.3 (9.4)	35.5 (**11.5**)	3RLJ	24.8 (0.0)	24.8 (3.1)	*27.9* (**11.5**)
	2AX9	28.4 (5.2)		2OZ7	25.5 (3.1)		
GR	3K22	29.3 (7.6)	28.3 (*4.7*)	1NHZ	29.5 (9.4)	29.6 (*4.7*)	**32.5** (9.4)
	6EL9	28.3 (4.7)		4MDD	24.3 (3.5)		
PR	1SQN	31.1 (15.8)	**34.0** (*13.0*)	2OVH	35.6 (8.8)	**39.2** (**10.6**)	**40.0** (*13.0*)
	3KBA	30.8 (8.8)		4OAR	32.9 (8.8)		
ERα	1X7R	32.9 (11.6)	31.0 (*7.6*)	1XP1	31.7 (12.6)	33.5 (*10.1*)	33.5 (12.6)
	5U2D	28.2 (8.4)		2IOK	30.2 (5.9)		
ERβ	3OLL	28.2 (8.1)	**31.7** (**9.2**)	1L2J	31.0 (11.7)	**34.2** (12.7)	**34.5** (11.7)
	1U3R	28.4 (8.1)		1NDE	31.1 (8.1)		
PPARα	2P54	21.2 (7.9)	27.8 (7.9)	1KKQ	29.6 (5.3)	29.6 (5.3)	30.1 (7.9)
	1I7G	27.4 (7.9)		2REW	26.6 (5.3)		
PPARγ	3BC5	20.9 (2.8)	21.0 (3.7)	5DWL	20.4 (2.8)	22.1 (4.4)	**23.6** (4.8)
	3VSO	18.2 (3.7)		5LSG	21.0 (4.6)		
RARα	3KMR	20.3 (0.0)	*21.4* (**2.4**)	1DKF	21.8 (2.4)	23.5 (2.4)	23.0 (2.4)
	3A9E	24.0 (0.0)		5K13	25.1 (2.4)		
RORγ	3LOL	20.1 (2.4)	20.8 (3.6)	5NTK	20.7 (1.2)	21.7 (**1.9**)	**23.9** (3.6)
	4WLB	21.0 (3.6)		5K3N	21.4 (1.2)		
VDR	3B0T	21.3 (1.8)	23.8 (**2.5**)	5XPL	20.4 (2.0)	21.3 (**2.4**)	22.9 (**2.5**)
	3AZ2	21.7 (2.2)		5XUQ	19.8 (1.4)		
RXRα	1MVC	20.7 (4.6)	**24.1** (4.6)	3NSQ	20.1 (3.1)	**27.6** (*7.7*)	**28.0** (9.2)
	5LYQ	21.5 (3.1)		2P1V	24.2 (9.2)		
LXRβ	1P8D	18.9 (3.1)	**21.3** (**5.2**)	6S4U	18.2 (1.0)	**21.2** (**4.2**)	**21.9** (**5.2**)
	3KFC	18.8 (4.2)		6S4N	19.0 (2.1)		

Consensus logAUC/EF1, logAUC/EF1 calculated from consensus docking scores from two structures of a single functional state (active or inactive state). Total consensus logAUC/EF1, logAUC/EF1 calculated from consensus docking scores from four structures (2 active and 2 inactive structures.) Consensus logAUC/EF1 (and Total consensus logAUC/EF1) is in bold/italic font when it is 10% larger/smaller than that from the best performing structure, and in a normal font in rest cases where it is considered to be comparable

### Consensus over multiple receptor structures

In virtual screening against single structures, the structural flexibility of the protein target and associated ligand selectivity are often not fully considered. To better take this into account, for each of 12 NRs, we included two active structures and two inactive structures. The consensus enrichments from 2 structures of each functional state and from 4 structures of two functional states were calculated separately.

#### Single functional state

First, for each NR, we compared the consensus enrichment (logAUC and EF1) over 2 receptor structures of a single functional state (either active or inactive) and the corresponding enrichments from single structure screening. Out of the 24 functional states of 12 NRs, consensus logAUC values are better, comparable to, and worse than that from the better performing structure in 33.3%, 62.5%, and 4.2% of cases, respectively; similarly, consensus EF1 values are better, comparable to, and worse than that from the best performing structure in 37.5%, 37.5% and 25.0% cases, respectively (Table 2). We note that the consensus enrichment values (both logAUC and EF1) in each of these worse cases, are still better than or comparable to that from the other structure in the same functional state.

#### Dual functional states

**Fig. 2:**
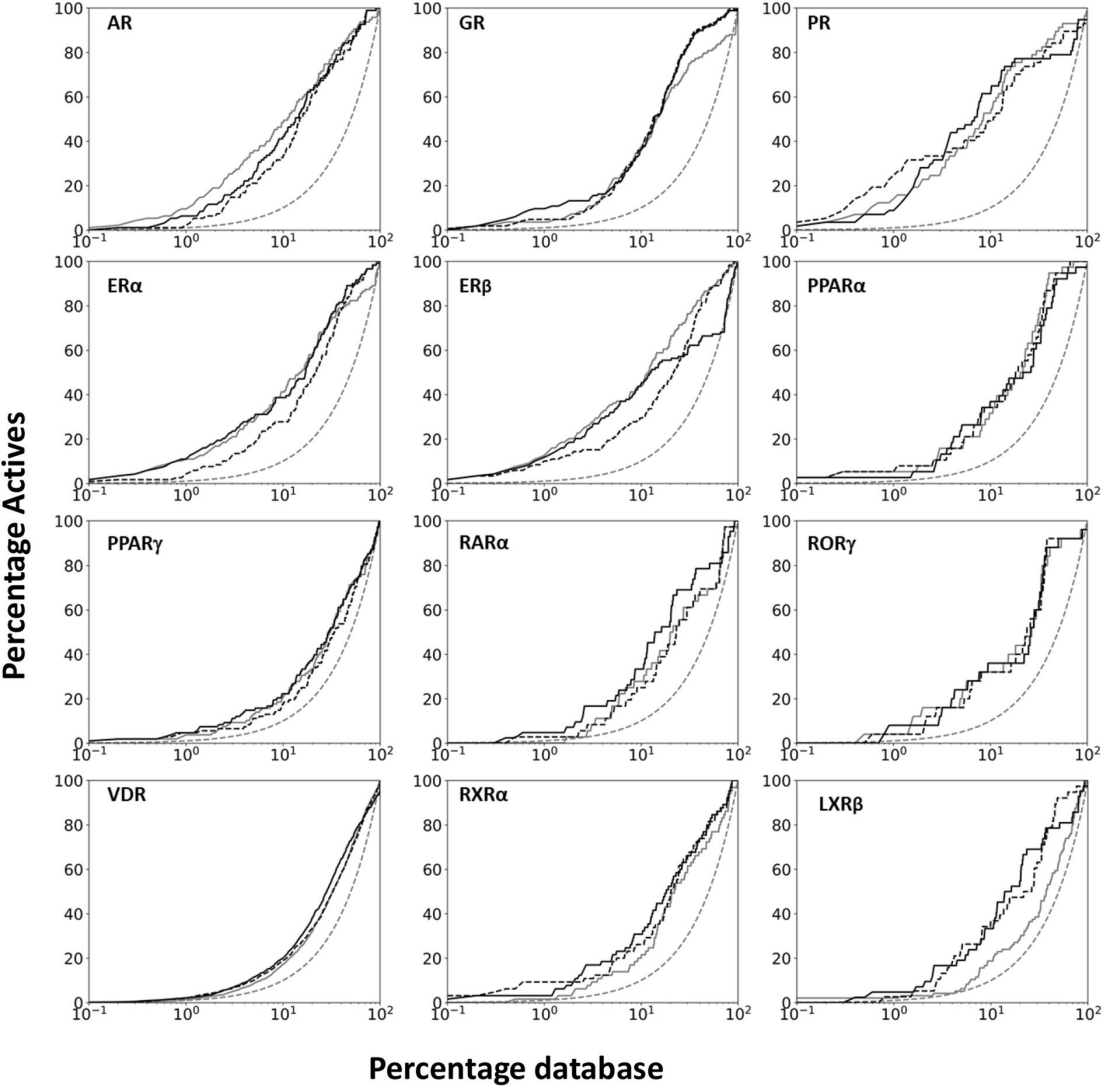
ToxCast active enrichment plots for 12 nuclear receptors, including random selection as reference (dotted line, grey), the enrichment from the best performing structure of each NR (solid line, grey), the consensus enrichment over 4 structures (2 active and 2 inactive structures) with *E*_DOCK_ (dotted line, black) and with *E'*_DOCK_ (solid line, black)

Second, for each NR, we calculated the total consensus enrichment (logAUC and EF1) over 4 receptor structures of both functional states. Out of 12 NRs, total consensus logAUC values are better than, comparable to, and worse than that from the best performing structure in 58.3%, 33.3%, and 8.3% of cases, respectively; similarly, total consensus EF1 values are better, comparable to, and worse than that from the best performing structure in 25.0%, 66.7% and 8.3% cases, respectively (Table 2; Fig. 2). We note that the total consensus enrichment values (both logAUC and EF1) in each of these worse cases are still better than or comparable to that from the second-best performing structure of that NR. When the consensus score over multiple structures was applied, the ranks of known EDCs were often improved in comparison to that from the best performing structure. For example, the rank of BPA against ERα and PPARγ has improved from 63 and 173 (the best performing structures) to 29 and 64, respectively; while the rank for DAP against ERα and PPARγ has improved from 254 and 128 to 88 and 67, respectively.

#### ToxCast active selectivity by different functional states

**Fig. 3:**
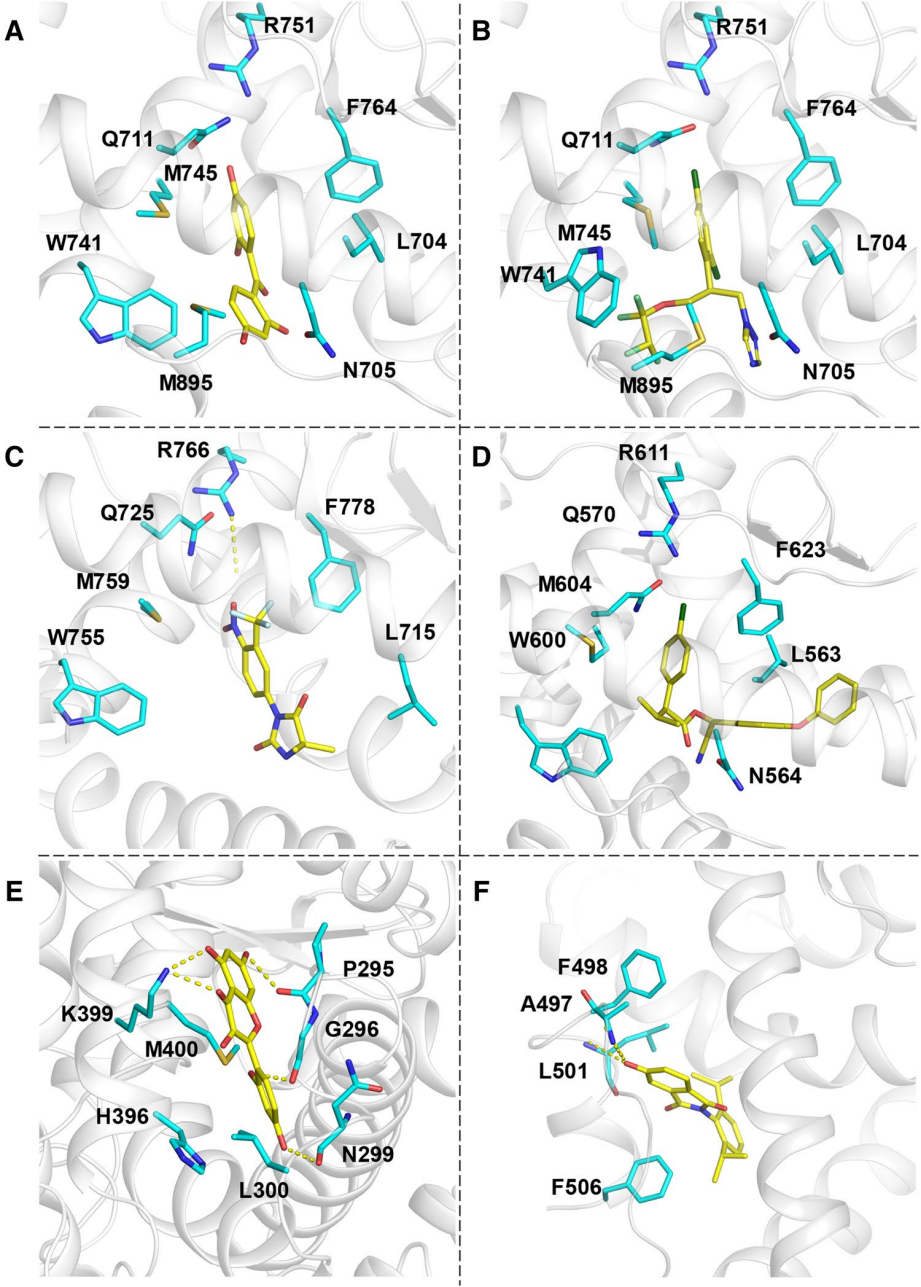
**a** Tetrahydroxybenzophenone docked in the active structure of AR (PDB ID 3L3X), **b** Triticonazole docked in the inactive structure of AR (PDB ID 2OZ7), **c** Nilutamide in PR (PDB ID: 1SQN), docking rank enhanced to 49 from 292 after applying the hybrid scoring scheme, **d** Esfenvalerate in GR (PDB ID: 4MDD), docking rank enhanced to 16 from 117 after applying the hybrid scoring scheme, **e** Morin, docked at alternate binding pocket 1 (ABP1) of PPARa (PDB ID: 2P54), **f** 5HPP-33, docked at alternate binding pocket 2 (ABP2) of RORγ (PDB ID: 5C4T)

To further understand whether docking against active or inactive structures confers any selectivity or not, we examined the docking screens in detail. Significant differences (10%) between consensus logAUC values from active and inactive structures were observed in AR, PR, RXRα, and RARα. In PR, RXRα, and RARα, inactive structures yielded higher consensus logAUC values than active structures; while in AR, it is the opposite—the consensus logAUC value from active structures (35.5) is higher than that from inactive structures (24.8). In AR and RXRα, we also observed a similar trend in consensus EF1 as their consensus logAUC. In AR, The significant differences in logAUC and EF1 are due to the fact that among ToxCast actives of AR, 38% of these ToxCast actives are known AR agonists (Kleinstreuer et al. 2017; Lynch et al. 2017). These observations suggest possible selectivity of ToxCast actives by active or inactive structures of these receptors. For instance, the ToxCast active tetrahydroxybenzophenone was ranked 14 in one active structure of AR (PDB ID: 3L3X, Fig. 3a) but only 669 in one inactive structure of the same receptor (PDB ID: 2OZ7). In contrast, the ToxCast active triconazole, was ranked 1565 and 4 in these two AR structures, respectively (Fig. 3b). We presume that these opposite trends may be due to conformational changes in TRP741, MET745, and MET895. In that active structure, these residues are stacked against each other, resulting in a smaller binding site that favors tetrahydroxybenzophenone over triconazole. Compared to their orientations in that active structure, these residues are further away from each other in that inactive structure, resulting in a larger binding site that favors triconazole over tetrahydroxybenzophenone.

These results on consensus ToxCast active enrichment are very promising, as simply by taking the consensus it will very likely approach the potential EDC recognition ability of the best or second-best performing structure that would often be difficult to know in advance in real applications. This could be because different structures of each receptor complement different groups of ToxCast actives but not the inactives so that a consensus selection relying on the best docking score of each chemical derived using multiple receptor structures from two functional states could rescue certain potential EDCs that would be missed by a single receptor structure.

### Hybrid scoring scheme

#### Enrichment based on chemical similarity index

**Fig. 4:**
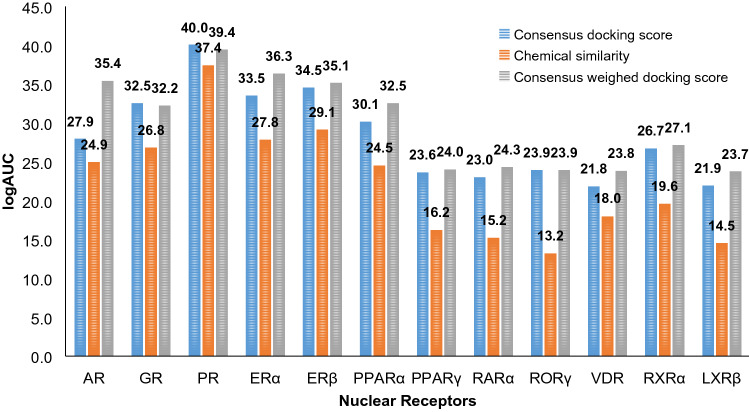
Comparison between ToxCast actives enrichment (logAUC) of chemical similarity-based screening (orange), consensus enrichment over 4 structures (2 active and 2 inactive structures) with *E*_DOCK_ (blue) and with *E'*_DOCK_ (grey)

In this study, we also performed virtual screening of 12 targets using the chemical similarity index (Tc, 2D fingerprints) of the chemicals with known binders (extracted from ChEMBL). We generated the Tc values with known binders from ChEMBL as mentioned in the methods section, then calculated their logAUC values based only on Tc values. The consensus logAUC values from 4 receptor structures using only Tc values of chemicals are worse than those using docking scores in 11 out of 12 NRs (91.6%), only comparable in the case of PR (Fig. 4). The performance of virtual screening using only Tc values, however, still yielded consensus enrichment better than random selection in 10 NRs. Since ligand-based (chemical similarity) and structure-based (docking) approaches are not completely correlated and both methods have reasonable enrichment potential (Ewing et al. 2006; Sastry et al. 2010; Kortagere et al. 2010; Huang et al. 2016; Cleves and Jain 2020), we explored an arbitrary combination of both approaches to enhance the overall enrichment. The assumption is that the respective errors in their enrichment are somewhat unlinked and that the integrated method may have a synergistic advantage.

#### Chemical similarity-weighted scoring scheme

To combine both chemical similarity and docking approaches, we implemented a hybrid scoring scheme (Eq. 1), resulting in a new score (*E'*_DOCK_) for each chemical. With our hybrid scoring scheme, improved and comparable logAUC values are observed in 39.6% and 60.4% of screening against the 48 structures used for the 12 NRs, with respect to those using original docking scores (*E*_DOCK_) (Table 3). For early enrichment (EF1), the hybrid scoring scheme led to an improvement in more cases (64.6%) and the rest remain comparable. Out of the 24 functional states of 12 NRs, the consensus logAUC of single functional state with *E'*_DOCK_ are better than and comparable to the logAUC with *E'*_DOCK_ from the more enriching structure in 29.2% and 70.8% of cases, respectively, while better than and comparable to the consensus logAUC of single functional state with *E*_DOCK_ in 25.0% and 75.0% of cases, respectively. Similarly, the consensus EF1 of single functional state with *E'*_DOCK_ are better than, comparable to, and worse than the EF1 with *E'*_DOCK_ from the more enriching structure in 25.0%, 41.7%, and 33.3% of cases, respectively, while better than and comparable to the consensus EF1 of single functional state with *E*_DOCK_ in 62.5% and 37.5% of cases, respectively. Considering all 4 receptor structures together, the total consensus logAUC with *E'*_DOCK_ are better than and comparable to the logAUC from the most enriching structure with *E'*_DOCK_ in 33.3% and 66.7% of cases, respectively, while better than and comparable to the total consensus logAUC with *E*_DOCK_ in 8.3% and 91.7% of cases, respectively. Similarly, the total consensus EF1 with *E'*_DOCK_ are better than, comparable to, and worse than the EF1 with *E'*_DOCK_ from the most enriching structure in 25.0%, 33.3%, and 41.7% of cases, respectively, while better than, comparable to, and worse than the total consensus EF1 with *E*_DOCK_ in 50.0%, 33.3%, and 16.7% of cases, respectively.

**Table 3:**
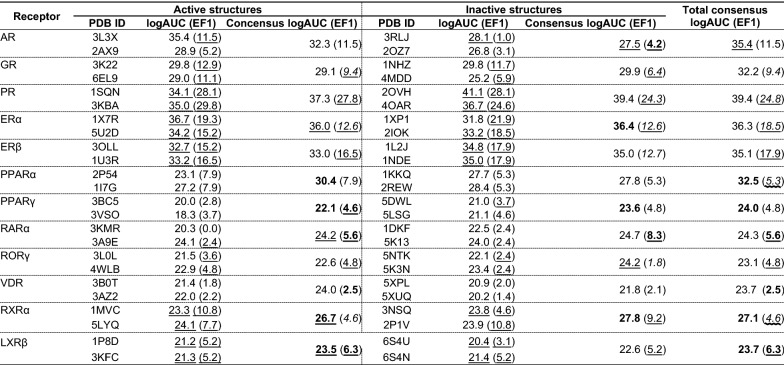
ToxCast active enrichment generated using the hybrid scoring scheme

logAUC/EF1, Consensus logAUC/EF1, and Total consensus logAUC/EF1 from the orthosteric pocket are calculated, compared, and marked in the same manner as in Table 2, except that the hybrid score (E'_DOCK_) is used in the calculation instead of the original docking score (E_DOCK_). These values are also compared to the counterparts in Table 2, and highlighted with straight or waved underline when they are better or worse than the counterparts in Table 2

Consensus logAUC/EF1 (and Total consensus logAUC/EF1) is in bold/italic font when it is 10% larger/smaller than that from the best performing structure, and in a normal font in rest cases where it is considered to be comparable

These comparisons between results from *E'*_DOCK_ and *E*_DOCK_ indicate that we can expect improved overall enrichment (logAUC) and more commonly improved early enrichment (EF1), without detrimental effects on the rest, when the hybrid scoring scheme is applied to docking results from single structures or from a consensus over multiple structures in the same functional state. For example, in the case of PR, the logAUC with *E'*_DOCK_ is improved by 10%, 14%, 15% and 12% in the 4 structures (PDB ID: 1SQN, 3KBA, 2OVH, and 4OAR), respectively; and the EF1 with *E'*_DOCK_ was improved by 78%, 239%, 219%, and 180%, respectively. These significant improvements are due to the fact that among ToxCast actives of PR, 37% of these ToxCast actives are chemically similar to the known ChEMBL ligands of PR (Tc ≥ 0.3), reflected by the improved ranks of these 57 chemicals with *E'*_DOCK_ (Table S3). In particular, the rank of a cancer drug Nilutamide (ToxCast active, T00001559, Tc = 0.49) is enhanced from 292 (with *E*_DOCK_) to 49 (with *E'*_DOCK_) against the active structure 1SQN (Fig. 3c). In the case of GR, the logAUC of the 4 structures (PDB ID: 3K22, 6EL9, 1NHZ, and 4MDD) are comparable to those with *E*_*DOCK*_. However, the EF1 with *E'*_DOCK_ was improved by 70%, 136%, 24%, and 68%, respectively. Among ToxCast actives of GR, only 18% of these ToxCast actives are chemically similar to the known ChEMBL ligands of GR. In particular, the rank of a pyrethroid insecticide Esfenvalerate (ToxCast active, T00001302, Tc = 0.69) is enhanced from 117 (with *E*_DOCK_) to 16 (with *E'*_DOCK_) against the inactive structure 4MDD (Fig. 3d). We also investigated the performance of known EDCs, when the hybrid scoring scheme was applied to the docking results, the scores and ranks were often improved. For example, the ranks based on total consensus scores of BPA against ERα and PPARγ are improved from 29 (with *E*_DOCK_) to 11 (with *E'*_DOCK_) and from 64 (with *E*_DOCK_) to 21 (with *E'*_DOCK_), respectively; and the ranks of DAP against ERα and PPARγ are improved from 88 (*E*_DOCK_) to 34 (with *E'*_DOCK_) and from 67 (with *E*_DOCK_) to 14 (with *E'*_DOCK_), respectively.

When the total consensus *E'*_DOCK_ (over all the 4 structures of each NR) was applied, at least 4 and 14 ToxCast actives are detected among the top-ranked 20 ToxCast chemicals (20% and 70%) for 10 and 5 NRs, respectively (Table S4). The numbers of ToxCast actives in the top-ranked 20 ToxCast chemicals using the total consensus *E'*_DOCK_, are higher than, equal to, and lower than those using the *E*_DOCK_ from the most enriching structures in 75.0%, 16.7%, and 8.3% of cases, respectively; while higher than, equal to, and lower than those using the total consensus *E*_DOCK_ in 50.0%, 33.3%, and 16.7% of cases, respectively. Considering the marginal but systematic improvement in ToxCast active enrichment, the combination of consensus over multiple structures and hybrid scoring scheme is probably the optimal method for potential EDC recognition, when both multiple receptor structures and known ligands are available for the target protein.

### Alternative pockets

Our previous results emphasized the importance of using multiple structures, hybrid scoring scheme in the recognition of ToxCast actives of 12 NRs. However, these calculations were done with the assumption that these actives bind only to the orthosteric binding pocket (OBP). However, AR (Lack et al. 2011; Lallous et al. 2016) and RORγ (Song et al. 2016) have been previously reported to have alternate binding pockets ABP1 and ABP2, respectively. In AR, the ABP1 (PDB ID: 2PIW) is also known as binding function 3 site (BF3), that is a hydrophobic site located at the junction of H1, the loop of H3-5, and H9, adjacent to activation function site (AF2, cofactor binding site). In RORγ, the ABP2 (PDB ID: 5C4T) consists of H4, H5, H11 and the repositioned H12 in its agonistic state, adjacent to the AF2 site but distal to OBP. Ligands binding to these ABPs have been implicated in the activation/inactivation mechanism in these nuclear receptors (Lack et al. 2011; Song et al. 2016; Lallous et al. 2016)*.* In this study, we extrapolated ABP1 of AR and ABP2 of RORγ to the best resolution structure of each of the remaining 11 NRs, respectively, because both ABP conformations vary little among the 4 receptor structures of each NR used in this study. Thereafter, ToxCast actives and inactives were docked to ABP1 and ABP2 of each NR.

11 and 3 docking screens at ABP1 (confirmed in AR, predicted in the rest 11 NRs) outperformed (≥ 10%) random and mediocore selection, respectively; while only 4 docking screens at ABP2 (confirmed in RORγ, predicted in the rest 11 NRs) outperformed random selection (Table 4). In comparison to the results from OBP, docking against ABP1 in 6 NRs yielded better (PPARα) or comparable (GR, PPARγ, RORγ, VDR, RXRα) performance and all the rest are worse. Combining results from OBP and ABP(s) together, the consensus logAUC of OBP + ABP1 are better than, comparable to, and worse than that from the logAUC of OBP in 25%, 58% and 17% of NRs, respectively; the consensus EF1 of OBP and ABP1 are better than, comparable to, and worse than that from the EF1 of OBP in 33%, 50% and 17% of NRs, respectively. The consensus logAUC and EF1 of OBP + ABP1 + ABP2 presented the same performance. These results suggest that consensus over OBP and ABP(s) may in *non-negligible* number of cases lead to reduced accuracy in ToxCast active prediction, therefore, caution should be taken for including ABP-targeted docking screens in the prediction of potential EDCs, especially when ABPs are only predicted. In the meantime, we note that in 8 NRs more than 10% ToxCast actives received better docking scores from ABP1 than those from OBP, with the highest ratio from PPARα (35%). These 8 NRs include 5 out of the 6 NRs that showed better ToxCast active enrichment from ABP1 than that from OBP, indicating ABP1 should be considered in chemical toxicity prediction at least for these 5 NRs (PPARα, GR, PPARγ, RORγ, RXRα). For example, in the case of PPARα, where the logAUC values from OBP and ABP1 are 21.2 and 28.5, respectively, and the consensus logAUC is enhanced to 32.6, three ToxCast actives including econazole, morin, and apomorphine, have much better docking scores (data not shown) and ranks from ABP1 (12, 15, and 61) than those from OBP (996, 445, and 1323), respectively. In ABP1, the docked morin formed hydrogen bond interactions with PRO295, GLY296, ASN299, and LYS399 using its hydroxy/keto groups, and pi–pi stacking interaction with HIS396 using its phenyl ring (Fig. 3e). In addition, docking screens against ABP(s) may still be beneficiary even when the enrichment values from ABP(s) are worse than those from OBP. For example, in the case of AR, the logAUC values from OBP are much better than those from ABP1, and only 7.0% of ToxCast actives have better docking scores from ABP1 than those from OBP (Table 4). However, ToxCast actives Nitrilotriacetic acid, lactofen, and bensulide received better docking scores (data not shown) and ranks (61, 88, 168) from ABP1 than those from OBP (231, 447, 884). Similarly, in the case of RORγ, the logAUC values from OBP are much better than those from ABP2, and only 4.8% of ToxCast actives have better docking scores from ABP2 than those from OBP. However, ToxCast actives 5HPP-33 (thalidomide derivative), parafuchsin, and reservertol received better docking scores (data not shown) and ranks (16, 38, 143) from ABP2 than those from OBP (1578, 1657, 808). In ABP2, the docked 5HPP-33 formed hydrogen bonding interactions with ALA497 and PHE498 using its hydroxy group (Fig. 3f). Similar receptor-ligand interactions can be seen in the RORγ crystal structure solved with bound ABP2 ligand (PDB ID: 5C4T).

**Table 4:** ToxCast active enrichment from allosteric pockets, and the consensus over different pockets

Receptor	logAUC (EF1) from OBP	logAUC (EF1) from ABP1	Consensus1 logAUC (EF1)	ToxCast actives% favoring ABP1	logAUC (EF1) from ABP2	Consensus2 logAUC (EF1)	ToxCast actives% favoring ABP2
AR*	35.3 (9.4)	22.4 (1.0)	*31.4* (**10.8**)	7.0	16.2 (1.0)	*31.3* (**10.8**)	6.1
GR	29.3 (7.6)	27.0 (5.3)	**32.6** (7.6)	19.0	15.4 (2.4)	**32.4** (7.6)	5.4
PR	31.1 (15.8)	27.1 (10.5)	33.1 (**17.6**)	12.5	19.2 (4.4)	33.2 (**17.6**)	6.2
ERα	31.7 (12.6)	23.3 (4.2)	*27.1* (12.6)	7.5	14.5 (2.1)	*27.2* (12.6)	3.6
ERβ	28.2 (8.1)	23.3 (2.8)	31.0 (**9.0**)	12.5	14.6 (2.8)	30.9 (**9.0**)	3.6
PPARα	21.2 (7.9)	28.5 (5.3)	**32.6** (7.9)	35.0	13.8 (2.7)	**32.4** (7.9)	4.0
PPARγ	21.0 (4.6)	21.0 (2.8)	22.6 (*3.7*)	21.6	15.6 (2.8)	**23.2** (*3.7*)	3.4
RARα	25.1 (2.4)	21.0 (4.8)	23.6 (**3.6**)	11.4	14.8 (2.4)	23.2 (**3.6**)	3.8
RORγ^#^	22.9 (4.8)	22.4 (3.6)	24.2 (*3.6*)	12.2	16.1 (1.2)	24.0 (*3.6*)	4.8
VDR	21.3 (1.8)	19.4 (2.4)	21.7 (1.8)	4.2	16.9 (1.1)	21.8 (1.8)	3.4
RXRα	20.7 (4.6)	22.2 (4.6)	**25.1** (4.6)	16.8	15.0 (2.3)	21.2 (4.6)	4.5
LXRβ	19.0 (2.1)	14.8 (0.0)	19.0 (2.1)	1.0	15.4 (2.1)	19.0 (2.1)	1.0

logAUC(EF1) from OBP, logAUC (EF1) from the orthosteric binding pocket in the structure of the best resolution. Consensus1 logAUC/EF1, Consensus logAUC/EF1 from OBP and ABP1. Consensus2 logAUC/EF1, Consensus logAUC/EF1 from OBP, ABP1, and ABP2. ToxCast actives% favoring ABP1, the percentage of ToxCAST actives that were scored better by ABP1 than OBP. Consensus1 logAUC/EF1 or Consensus2 logAUC/EF1 are highlighted in bold or italic fonts when they are > 10% better or worse than logAUC (EF1) from OBP

*For AR, the reported alternative binding pocket (ABP1) (PDB ID: 2PIW) was used for docking, the ABP1 in each of the other 11 NRs was derived by superposing the best resolution structure onto 2PIW

^#^For RORγ, the reported alternative binding pocket (ABP2) (PDB ID: 5C4T) was used for docking, the ABP2 in each of the other 11 NRs was derived by superposing the best resolution structure onto 5C4T

Consensus1 logAUC/EF1 or Consensus2 logAUC/EF1 are highlighted in bold or italic fonts when they are >10% better or worse than logAUC (EF1) from OBP

### Prediction of novel fatty acids binding to PPARγ

PPARγ is one of the three PPAR isoforms (α, β, and γ). It is involved in transcriptional regulation of glucose and lipid metabolism (Yu et al. 1995; Lemberger et al. 1996; Desvergne and Wahli 1999; Tyagi et al. 2011), and mainly regulates adipose differentiation. Numerous natural endogenous and dietary lipids and their metabolites act as PPARγ activators, including polyunsaturated fatty acids (PUFAs) such as docosahexaenoic acid (DHA), and eicosapentanoic acid (EPA). The PUFAs and saturated fatty acids play important roles in membrane structure, bioactive compound production, and cellular signaling processes. Nowadays, they are increasingly consumed as food additives and supplements, while the consumption of saturated fats and unsaturated fats is considered to be harmful and beneficial, respectively. Therefore, it is important to recognize dietary fatty acids that can activate PPARγ through binding. Such discovery is very useful for the hazard or risk assessments of food products and prioritize specific food components and/or additives for further experimental assessments.

In the benchmarking of ToxCast chemicals, the enrichment of PPARγ actives using four PPARγ structures and the hybrid scoring function *E'*_DOCK_ is only 24.0 for logAUC and 4.8 for EF1 (Table 3). To facilitate PPARγ ligand identification out of fatty acids, we attempted to improve the existing docking-based potential EDC prediction method, following the same philosophy that consensus enrichment of more structures will be better than or comparable to that of fewer structures. Given the 222 crystal structures determined for PPARγ, we added two more agonist-bound structures and two more antagonist-bound structures to the PPARγ structure dataset, in total 8 structures that were solved in complex with drug-like molecules but not fatty acids. When the ToxCast library was screened against these 8 structures with *E'*_DOCK_ (only considering OBP), a better ToxCast active enrichment was achieved (logAUC = 28.9, EF1 = 7.4) (Table S5). The two EDCs BPA and DAP, that are known to bind and activate PPARγ, are now ranked 9 and 11 (previously ranked 11 and 14) out of the 1768 ToxCast chemicals, respectively. These results pushed the upper limit of the prediction accuracy of our computational method, suggesting again the beneficial effect of the usage of multiple structures.

#### Fatty acid docking analysis

Thereafter, we applied this improved method to predict dietary fatty acids that bind to PPARγ and likely activate the receptor. A fatty acid database of 252 fatty acids was screened against the 8 structures of PPARγ. In the top-ranked 25 fatty acids, we found that the fatty acids belong to 5 classes, including 8 furan fatty acids, 2 cyclopropyl fatty acids, 5 oxo fatty acids, 3 very long-chain fatty acids, and 7 PUFAs (Table S7). These fatty acids are well accommodated in the manner similar to known binders such as DHA and EPA. Based on *E'*_DOCK_ scores and chemical diversity, 7 unknown fatty acids belonging to different fatty acids classes were shortlisted for testing from top-ranked 25 fatty acids, including 2 furan fatty acids (furannonanoic acid (FNA), and furanundecanoic acid (FUA)), 1 cyclopropyl fatty acid (phytomonic acid (PTA)), 1 oxo fatty acid (ricinoleic acid (ROA)), 3 PUFAs (eicosatrienoic acid (ESA), pinolenic acid (PLA), and docosapentaenoic acid (DPA)). The very long-chain fatty acids are commercially not available so we did not select them. These 7 unknown fatty acids and 2 known PPARγ binders/activators (eicosapentaenoic acid (EPA) and docosahexaenoic acid (DHA)) as control were selected for testing, in total 9 fatty acids.

**Fig. 5:**
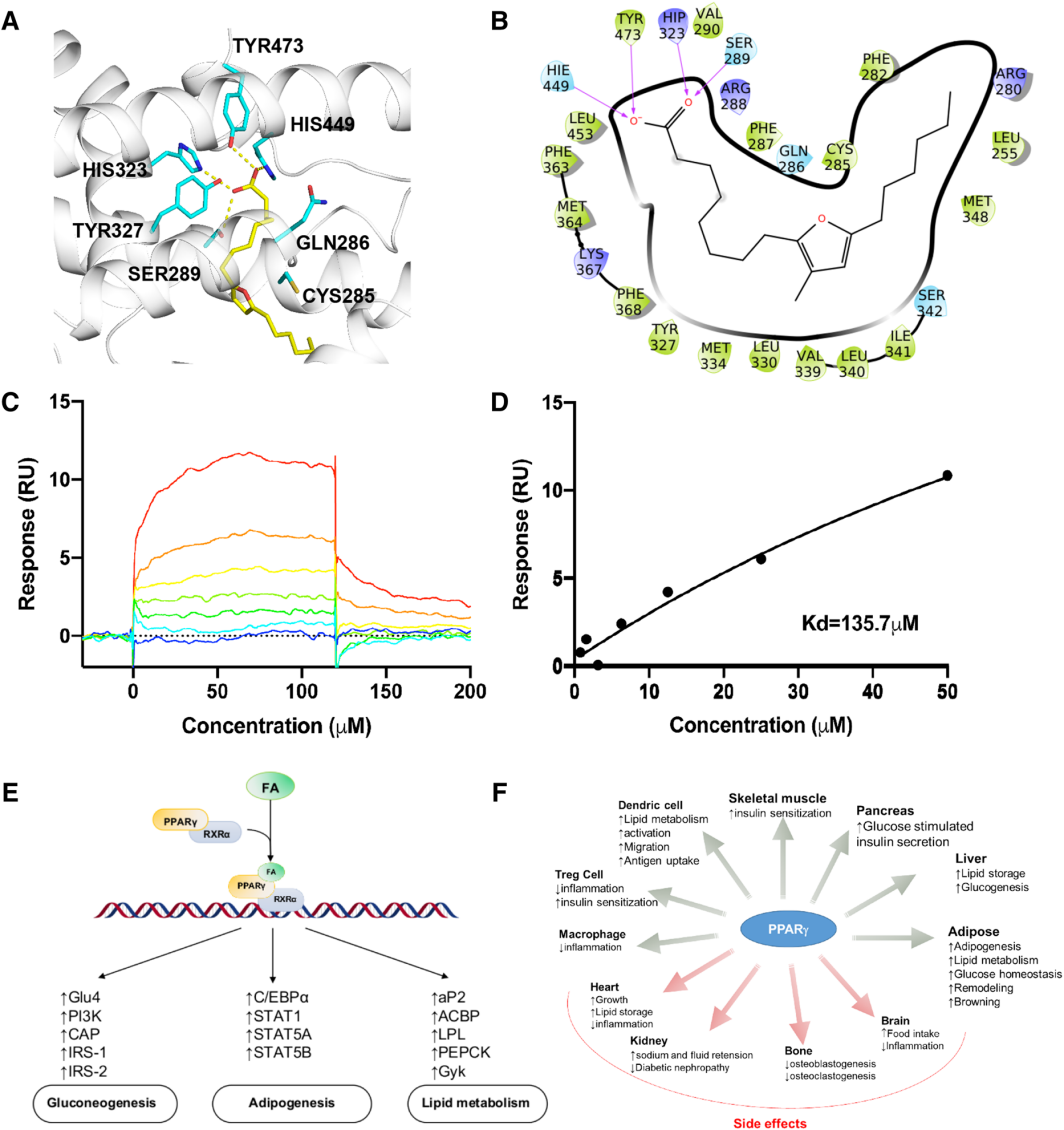
**a** Binding pose of FNA at PAPRγ binding site, **b** 2D FNA-PPARγ interaction diagram, **c** Surface plasmon resonance (SPR) assay of specific binding affinities of FNA to immobilized PPARγ on the CM5 sensor chip surface, sensorgram overlay and **d** equilibrium binding curve of FNA to PPARγ LBD, **e** Functional patterns of PPARγ, involved in adipocyte differentiation, lipid metabolism and glucose homeostasis, **f** Pathophysiology effects of PPARγ

To date, 28 crystal structures of PPARγ were solved in complex with fatty acids. Structure analysis showed that the carboxyl group in these fatty acids can form hydrogen bonds with SER289, HIS323, HIS449, and TYR473, which are reported to be vital for producing the maximum activity of compound through a direct stabilization of helix H12 and are responsible for the PPARγ transactivation activity (Itoh et al. 2008; Farce et al. 2009; Guasch et al. 2011). In addition, these fatty acids also form close contacts with PHE282, CYS285, GLN286, ARG288, VAL290, GLU295, LYS319, TYR327, MET334, VAL339, SER342, TYR355, PHE 363, MET364, LYS367, and LEU453 (hot-spots). The docking poses of the 9 shortlisted fatty acids show similar binding patterns as those observed in the 28 crystal structures. For example, the terminal carboxyl group of FNA forms hydrogen bonding interactions with SER289, HIS323, TYR327 and TYR473, and its unsaturated chain forms hydrophobic interactions with PHE282, CYS285, GLN286, MET364 and LEU453 (Fig. 5a and b). The docking scores of FNA, FUA, and PTA are better than those of DPA, ESA, PLA, and ROA probably because the furan rings in FNA and FUA, and the cyclopropane ring in PTA form more favorable hydrophobic interactions with CYS285, MET364, and VAL339 in comparison to the linear chains in other four fatty acids (Table 5).

**Table 5:** Docking and binding assay data for 9 tested fatty acids

No.	Fatty acids	Hybrid score	Kd (μM)	Rmax (RU)	Chi^2^
1.	Furannonanoic acid (FNA)	− 11.19	135.7	38.55	0.62
2.	Furanundcanoic acid (FUA)*	− 11.16	248.7	459.3	12.5
3.	Docosahexaenoic acid (DHA)	− 11.14	593	281	1.14
4.	Eicosapentaenoic acid (EPA)	− 10.12	629.4	136	1.27
5.	Doceapentaenoic acid (DPA)	− 10.10	3960	2167	0.08
6.	Eicosatrienoic acid (ESA)	− 10.01	> 10,000.00	> 10,000.00	3.19
7.	Phytomonic acid (PTA)	− 9.89	102.9	33.28	0.1
8.	Ricinoleic acid (ROA)	− 9.70	> 10,000.00	> 10,000.00	0.16
9.	Pinolenic acid (PLA)	− 9.27	> 10,000.00	> 10,000.00	0.74

*Chip coupled with 12000RU ligand (PPARγ LBD) was used for FUA binding analysis. *RU* response units. Chi^2^ is a measure of the average deviation of the experimental data from the fitted curve. Lower Chi^2^ values indicate a better fit; Rmax, the amount of ligand (in RUs) immobilized

#### PPARγ binding verification

We tested the binding of fatty acids to PPARγ through SPR analysis. As predicted, all the tested fatty acid exhibited weak binding to PPARγ with the calculated affinities (Kd) ranging from micromole to millimole. Specifically, EPA and DHA, which have been reported to activate PPARγ (Xu et al. 1999), showed a similar binding affinity with Kds of ~ 600 µM. The FNA (Fig. 5c and d), FUA, and PTA exhibited stronger binding than DHA and EPA (Fig. S1), while DPA, ESA, PLA and ROA showed weaker binding than DHA, which was well supported by their docking scores (Table 5). The higher binding affinity from PTA, FNA, and FUA are likely due to presence of a hydrophobic carbon ring (furan in FNA and FUA, cyclopropane in PTA) that forms more favorable hydrophobic interactions with CYS285, MET364, VAL339, and LEU453 compared to other four linear chain fatty acids, and the lower affinities of DPA, ESA, PLA and ROA are probably due to reduced carbon chain or degree of saturation.. Long-chain fatty acids (LCFA), such as DHA and EPA, have been identified as endogenous ligands of PPARγ and can regulate the lipid metabolism through modulating the transcriptional activity of PPARγ (Xu et al. 1999; Itoh et al. 2008). However, the LCFA can be pushed out of the orthosteric pocket by synthetic ligands, as their affinities are much lower than synthetic ligands (Itoh et al. 2008). This notion agrees well with our result that the LCFAs displayed weak binding to PPARγ in molecular docking and SPR assays. Mutations in these fatty acid-binding residues have been found to be associated with various metabolic and inflammatory diseases, such as obesity (Ristow et al. 1998; Beamer et al. 1998), Insulin resistance, diabetes (Barroso et al. 1999; Majithia et al. 2014), hypertension (Barroso et al. 1999), lipodystrophy (Francis et al. 2006; Miehle et al. 2016), dyslipidaemia (Capaccio et al. 2010), colon and bladder cancer (Sarraf et al. 1999; Liu et al. 2019). For example, the V290M mutation is associated with severe insulin resistance, diabetes mellitus, and hypertension (Fig. 5e, f) (Barroso et al. 1999). This mutant inhibits PPARγ function in a dominant-negative manner, markedly attenuating the transcriptional function of PPARγ. Other PPARγ-deficient hot-spot residue mutations in the fatty acid-binding site are associated with colon cancer such as Q286P, K319X, R288H/A, S289C (Sarraf et al. 1999) and with lipodystrophy (Francis et al. 2006; Miehle et al. 2016) such as Y355X, Y473A, R165T and L339X. For two of these mutants Y355A and V290M, we constructed their structures using the “mutate” option present in Maestro, Schrödinger, and attempted to estimate the binding affinity of the selected fatty acids against these two mutants using docking analysis. The results showed that all the 9 tested fatty acids consistently received worse docking scores from mutant structures with respect to their docking scores from the wild type structure. For example, FNA received a docking score of − 11.19 from wild type structure but only − 8.65 and − 9.31 from Y355A and V290M mutant structures, respectively (Table S8). These results suggest that we may expect weaker binding of these fatty acids to the PPARγ mutants Y355A and V290M, and subsequently reduced transcriptional activity with respect to that of the wild type PPARγ. The long-term malfunction of PPARγ may lead to an increased risk of metabolic disorders such as insulin resistance, diabetes mellitus, or partial lipodystrophy (Barroso et al. 1999; Francis et al. 2006).

#### Confirmed fatty acids in diets

Among the fatty acids that exhibited higher affinity to PPARγ than endogenous ligands such as DHA and EPA, FNA (9M5) was reported to enhance adipogensis (Lauvai et al. 2019), and to promote significantly more lipid accumulation than PUFAs like DHA and EPA even at lower concentrations, consistent with our results. However, it is currently unknown if FNA can directly interact with and activate PPARγ. Our study provides a plausible mechanism that can explain the observed phenotypic effects of FNA. Due to its strong binding affinity experimentally verified in our study, FNA could outcompete natural activators such as oelic, linoleic, lauric and arachidonic acid upon binding to PPARγ. The same behaviour could be observed in related analogues of FNA and FUA (Table S6) such as 9D5, 11D5 and 7D5. These furan fatty acids are primarily found in a wide range of diets such as fish liver oil (at the level of 1–6%) and freshwater fish liver oil (up to 25%), plant oils, fruits (e.g. lemon, olives, strawberry), vegetables (e.g. cabbage and potato), and mushrooms (Spiteller 2005; Xu et al. 2017). Similarly, PTA as a stronger binder of PPARγ, than DHA and EPA, is also found in various dietary products such as milk (Caligiani et al. 2014), rapseed oil (Berdeaux et al. 2010), mushrooms, and probiotics (e.g. lactobacillus lipids) (Karine Pedneault et al. 2006; Nandakumar and Tan 2008). Although PTA is saturated fatty acid, it can be highly reactive because of its highly strained cyclopropane ring at 11, 12-position. In particular, strained cyclopropane rings can react with thiol/sulfur groups (i.e. with active cysteine residues in receptors). In PPARγ active site, we noticed a reactive cysteine (CYS285), which can form covalent bond with the strained cyclopropane ring of PTA, like 15d-PGJ2 (endogenous ligand), which activates PPARγ through covalent bond formation with CYS285 (Liberato et al. 2012). This could partially account for the relatively high affinity of PTA against PPARγ. Other fatty acids chemically similar to PTA could resemble its strong interaction against PPARγ, such as dihydro sterculic acid and cyclopropenoic acid derivatives (e.g. sterculic acid and malvalic acid). Dihydrosterculic acid and sterculic acid are available in sterculia foetida (~ 50%) and cotton-seed oil (2%). The sterculia foetida oil diet for rats showed healthy effects such as reduced reproductive function, retarted growth, and weight gain (Nixon et al. 1974; Eisele et al. 1977; Matlock et al. 1985; Peláez et al. 2020). Similar impact can be anticipated for PTA as it is chemically similar to sterculic acid and shows high binding affinity against PPARγ. Among the fatty acids we selected, DPA is also confirmed to bind to PPARγ, weaker than DHA and EPA. DPA is a ω-3 fatty acid that belongs to PUFA family; it is a metabolic product of the parent fatty acid is α-linolenic acid.

## Conclusions

### Overview

In this study, we constructed an in silico method for potential EDC prediction, based on molecular docking. This method, when evaluated on 12 nuclear receptors (NRs), showed the reasonable capability to recognize ToxCast actives out of inactives (logAUC values range from 21.8 to 39.4, EF1 values range from 1.8 to 24.3, see Table 3), nearly 2 to 10 times better than random selection. These results suggest aspects to better exploit structure-based approaches in potential EDC prediction: consensus over docking screens against multiple protein structures, chemical similarity-weighted docking scores, alternative binding sites (ABPs).

### Consensus over multiple receptor structures

Consensus enrichment scores over multiple receptor structures covering both agonist-bound and antagonist-bound states, computed by selecting the best docking score for each chemical, can perform consistently better than or comparable to best-performing single structure (Table 2). When the performance of each crystal structure in EDC recognition is not known, the consensus selection over multiple receptor structures is the most suitable method for potential EDC prediction.

### Hybrid scoring including ligand information

The hybrid (chemical similarity-weighted) scoring approach can perform better than both the protein-based (docking) approach and the ligand-based (chemical similarity) approach (Table 3; Fig. 4), when applied to results from every single structure, and to that from consensus over multiple structures. The proposed method, combining consensus over multiple structures and hybrid scoring scheme, could be an optimal approach for potential EDC prediction, when both protein structure and ligand information are available for the target.

### Alternative binding site

ToxCast actives can be better scored (from 1 to 35%) by ABPs than the orthosteric binding site (OBP) (Table 4), therefore, we recommend to take ABPs into account in potential EDC prediction especially when ABPs have been verified by experiments. However, it may not be straightforward to use the consensus over OBP and ABP(s) in the high-throughput in silico prediction, as reduced accuracy are observed in some cases.

### Fatty acids identified as PPARγ ligands

The in silico method was applied to the identification of novel fatty acids binding to PPARγ, which is a key protein in transcriptional regulation of glucose and lipid metabolism, and involved in various metabolic and inflammatory diseases. A total of 7 fatty acids were predicted as ligand (likely activator) candidates, 4 out of which were verified by subsequent binding tests, including 3 fatty acids (FNA, FUA and PTA) of better binding affinity (Kd = 100–250 μM) than DHA and EPA. Mutations of binding site residues of these fatty acids have been found in cancer, diabetes, and hypertension.

### Future perspectives

In silico prediction of chemical toxicity and understanding the molecular initiating events remain major challenges in toxicology. In this study, we showed on 12 NRs that a virtual screening method based on molecular docking can contribute to addressing these challenges. The utility of this method can be enlarged by including all 45 NRs that have at least one crystal structure solved (out of 48 identified NRs) (Lagarde et al. 2014; Weikum et al. 2018). In addition, only the ligand-binding domains (LBDs) of NRs were targeted in this study. However, it has been suggested that the DNA binding domains (DBDs) of NRs and even the DNA sites recognized by NRs can also be targeted by ligands (Brodie 2005; Meijsing et al. 2009; Li et al. 2014; Dalal et al. 2014; Shizu et al. 2018; Frank et al. 2018; Pal et al. 2019; Veras Ribeiro Filho et al. 2019). Thus we expect that the application of our in silico method to these new molecular interfaces will also facilitate potential EDC prediction. Subsequent in vitro assessments may be performed to better understand the toxicodynamics of these suspected EDCs, establish their concentration-dependent effects to cellular functions, and assess if they may pose acceptable or unacceptable risks to the relevant exposed human populations.

## Electronic supplementary material

Below is the link to the electronic supplementary material.

Supplementary file 1 (DOCX 3606 kb)

## Abbreviations

NR: Nuclear receptor
AR: Androgen receptor
ER: Estrogen receptor
GR: Glucocorticoid receptor
PPAR: Peroxisome proliferator-activated receptor
PR: Progesterone receptor
RAR: Retinoic acid receptor
RXR: Retinoic X receptor
VDR: Vitamin D receptor
ROR: RAR-related orphan receptor
LXR: Liver X receptor
EDC: Endocrine-disrupting chemical
US-EPA: United States environmental protection agency
PUFA: Polyunsaturated fatty acids
HTS: High-throughput in vitro screening
AUC: Area under the curve
RMSD: Root mean square deviation

## References

[CR1] Bain DL, Heneghan AF, Connaghan-Jones KD, Miura MT (2007). Nuclear receptor structure: implications for function. Annu Rev Physiol.

[CR2] Barroso I, Gurnell M, Crowley VEF (1999). Dominant negative mutations in human PPARγ associated with severe insulin resistance, diabetes mellitus and hypertension. Nature.

[CR3] Beamer BA, Yen CJ, Andersen RE (1998). Association of the Pro12Ala variant in the peroxisome proliferator-activated receptor-gamma2 gene with obesity in two Caucasian populations. Diabetes.

[CR4] Berdeaux O, Gregoire S, Fournier C (2010). Detection of lactobacillic acid in low erucic rapeseed oil—A note of caution when quantifying cyclic fatty acid monomers in vegetable oils. Chem Phys Lipids.

[CR5] Bordoni A, Di Nunzio M, Danesi F, Biagi PL (2006). Polyunsaturated fatty acids: from diet to binding to ppars and other nuclear receptors. Genes Nutr.

[CR6] Brodie J (2005). Intra-domain communication between the N-terminal and DNA-binding domains of the androgen receptor: modulation of androgen response element DNA binding. J Mol Endocrinol.

[CR7] Caligiani A, Marseglia A, Palla G (2014). An overview on the presence of cyclopropane fatty acids in milk and dairy products. J Agric Food Chem.

[CR8] Canvas, Schrödinger, LLC, New York, NY 2018

[CR9] Capaccio D, Ciccodicola A, Sabatino L (2010). A novel germline mutation in peroxisome proliferator-activated receptor γ gene associated with large intestine polyp formation and dyslipidemia. Biochim Biophys Acta Mol Basis Dis.

[CR10] Capuzzi SJ, Politi R, Isayev O (2016). QSAR modeling of Tox21 challenge stress response and nuclear receptor signaling toxicity assays. Front Environ Sci.

[CR11] Carlson H (2002). Protein flexibility is an important component of structure-based drug discovery. Curr Pharm Des.

[CR12] Cavasotto C, Singh N (2008). Docking and high throughput docking: successes and the challenge of protein flexibility. Curr Comput Aided-Drug Des.

[CR13] Cleves AE, Jain AN (2020). Structure- and ligand-based virtual screening on DUD-E + : performance dependence on approximations to the binding pocket. J Chem Inf Model.

[CR14] Cohen Hubal EA, Richard A, Aylward L (2010). Advancing exposure characterization for chemical evaluation and risk assessment. J Toxicol Environ Heal Part B.

[CR15] Cozzini P, Kellogg GE, Spyrakis F (2008). Target flexibility: an emerging consideration in drug discovery and design †. J Med Chem.

[CR16] Dalal K, Roshan-Moniri M, Sharma A (2014). Selectively targeting the DNA-binding domain of the androgen receptor as a prospective therapy for prostate cancer. J Biol Chem.

[CR17] Desvergne B, Wahli W (1999). Peroxisome proliferator-activated receptors: nuclear control of metabolism. Endocr Rev.

[CR18] Diamanti-Kandarakis E, Bourguignon J-P, Giudice LC (2009). Endocrine-disrupting chemicals: an endocrine society scientific statement. Endocr Rev.

[CR19] Dix DJ, Houck KA, Martin MT (2007). The ToxCast program for prioritizing toxicity testing of environmental chemicals. Toxicol Sci.

[CR20] Eisele TA, Yoss JK, Nixon JE (1977). Rat urinary metabolites of [9,10-methylene-14C]sterculic acid. Biochim Biophys Acta Lipids Lipid Metab.

[CR21] European Council (2006) European Commision. REGULATION (EC) No 1907/2006 OF THE EUROPEAN PARLIAMENT AND OF THE COUNCIL of 18 December 2006 concerning the Registration, Evaluation, Authorisation and Restriction of Chemicals (REACH), establishing a European Chemicals Agency, amend

[CR22] Ewing T, Baber JC, Feher M (2006). Novel 2D fingerprints for ligand-based virtual screening. J Chem Inf Model.

[CR23] Fan H, Irwin JJ, Sali A (2012). Computational drug discovery and design.

[CR24] Fan H, Irwin JJ, Webb BM (2009). Molecular docking screens using comparative models of proteins. J Chem Inf Model.

[CR25] Farce A, Renault N, Chavatte P (2009). Structural insight into PPARγ ligands binding. Curr Med Chem.

[CR26] Foster JM, Moreno P, Fabregat A (2013). LipidHome: a database of theoretical lipids optimized for high throughput mass spectrometry lipidomics. PLoS ONE.

[CR27] Fradera X, de la Cruz X, Silva CHTP (2002). Ligand-induced changes in the binding sites of proteins. Bioinformatics.

[CR28] Francis GA, Li G, Casey R (2006). Peroxisomal proliferator activated receptor-γ deficiency in a Canadian kindred with familial partial lipodystrophy type 3 (FPLD3). BMC Med Genet.

[CR29] Frank F, Okafor CD, Ortlund EA (2018). The first crystal structure of a DNA-free nuclear receptor DNA binding domain sheds light on DNA-driven allostery in the glucocorticoid receptor. Sci Rep.

[CR30] Glide, Schrödinger, LLC, New York, NY 2018

[CR31] Guasch L, Sala E, Valls C (2011). Structural insights for the design of new PPARgamma partial agonists with high binding affinity and low transactivation activity. J Comput Aided Mol Des.

[CR32] https://lipidbank.jp/

[CR33] https://www.ebi.ac.uk/chembl/

[CR34] https://www.epa.gov/chemical-research/toxicity-forecaster-toxcasttm-data ToxCast

[CR35] https://www.rcsb.org/

[CR36] Huang P, Chandra V, Rastinejad F (2010). Structural overview of the nuclear receptor superfamily: insights into physiology and therapeutics. Annu Rev Physiol.

[CR37] Huang SY, Li M, Wang J, Pan Y (2016). HybridDock: a hybrid protein-ligand docking protocol integrating protein- and ligand-based approaches. J Chem Inf Model.

[CR38] Hussain F, Basu S, Heng JJH (2020). Predicting direct hepatocyte toxicity in humans by combining high-throughput imaging of HepaRG cells and machine learning-based phenotypic profiling. Arch Toxicol.

[CR39] Itoh T, Fairall L, Amin K (2008). Structural basis for the activation of PPARγ by oxidized fatty acids. Nat Struct Mol Biol.

[CR40] Judson R, Richard A, Dix D (2008). ACToR—Aggregated computational toxicology resource. Toxicol Appl Pharmacol.

[CR41] Judson R, Richard A, Dix DJ (2009). The toxicity data landscape for environmental chemicals. Environ Health Perspect.

[CR42] Karine PEDNEAULT, Paul ANGERS, André GOSSELIN, Russell JTWEDDELL (2006). Fatty acid composition of lipids from mushrooms belonging to the family Boletaceae. Mycol Res.

[CR43] Kavlock R, Chandler K, Houck K (2012). Update on EPA’s ToxCast program: providing high throughput decision support tools for chemical risk management. Chem Res Toxicol.

[CR44] Kersten S, Desvergne B, Wahli W (2000). Roles of PPARs in health and disease. Nature.

[CR45] Kitchen DB, Decornez H, Furr JR, Bajorath J (2004). Docking and scoring in virtual screening for drug discovery: methods and applications. Nat Rev Drug Discov.

[CR46] Kleinstreuer NC, Ceger P, Watt ED (2017). Development and validation of a computational model for androgen receptor activity. Chem Res Toxicol.

[CR47] Kliewer SA, Sundseth SS, Jones SA (1997). Fatty acids and eicosanoids regulate gene expression through direct interactions with peroxisome proliferator-activated receptors and. Proc Natl Acad Sci.

[CR48] Knegtel RM, Kuntz ID, Oshiro CM (1997). Molecular docking to ensembles of protein structures. J Mol Biol.

[CR49] Knudsen TB, Houck KA, Sipes NS (2011). Activity profiles of 309 ToxCast^TM^ chemicals evaluated across 292 biochemical targets. Toxicology.

[CR50] Kortagere S, Krasowski MD, Reschly EJ (2010). Evaluation of computational docking to identify pregnane X receptor agonists in the ToxCast database. Environ Health Perspect.

[CR51] Lack NA, Axerio-Cilies P, Tavassoli P (2011). Targeting the binding function 3 (BF3) site of the human androgen receptor through virtual screening. J Med Chem.

[CR52] Lagarde N, Ben Nasr N, Jérémie A (2014). NRLiSt BDB, the manually curated nuclear receptors ligands and structures benchmarking database. J Med Chem.

[CR53] Lallous N, Leblanc E, Munuganti RSN (2016). Targeting binding function-3 of the androgen receptor blocks its co-chaperone interactions, nuclear translocation, and activation. Mol Cancer Ther.

[CR54] Lauvai J, Becker A, Lehnert K (2019). The Furan fatty acid 9M5 acts as a partial ligand to peroxisome proliferator-activated receptor gamma and enhances adipogenesis in 3T3-L1 preadipocytes. Lipids.

[CR55] Lee J-YJ, Miller JA, Basu S (2018). Building predictive in vitro pulmonary toxicity assays using high-throughput imaging and artificial intelligence. Arch Toxicol.

[CR56] Lemberger T, Desvergne B, Wahli W (1996). Peroxisome proliferator-activated receptors: a nuclear receptor signaling pathway in lipid physiology. Annu Rev Cell Dev Biol.

[CR57] Li H, Ban F, Dalal K (2014). Discovery of small-molecule inhibitors selectively targeting the DNA-binding domain of the human androgen receptor. J Med Chem.

[CR58] Liberato MV, Nascimento AS, Ayers SD (2012). Medium chain fatty acids are selective peroxisome proliferator activated receptor (PPAR) γ activators and pan-PPAR partial agonists. PLoS ONE.

[CR59] LigPrep, Schrödinger, LLC, New York, NY 2018

[CR60] Lim VJY, Du W, Chen YZ, Fan H (2018). A benchmarking study on virtual ligand screening against homology models of human GPCRs. Proteins Struct Funct Bioinforma.

[CR61] Liu C, Tate T, Batourina E (2019). Pparg promotes differentiation and regulates mitochondrial gene expression in bladder epithelial cells. Nat Commun.

[CR62] Lynch C, Sakamuru S, Huang R (2017). Identifying environmental chemicals as agonists of the androgen receptor by using a quantitative high-throughput screening platform. Toxicology.

[CR63] Ma B, Shatsky M, Wolfson HJ, Nussinov R (2009). Multiple diverse ligands binding at a single protein site: a matter of pre-existing populations. Protein Sci.

[CR64] Madrazo JA, Kelly DP (2008). The PPAR trio: regulators of myocardial energy metabolism in health and disease. J Mol Cell Cardiol.

[CR65] Majithia AR, Flannick J, Shahinian P (2014). Rare variants in PPARG with decreased activity in adipocyte differentiation are associated with increased risk of type 2 diabetes. Proc Natl Acad Sci.

[CR66] Manco M, Calvani M, Mingrone G (2004). Effects of dietary fatty acids on insulin sensitivity and secretion. Diabetes Obes Metab.

[CR67] Marion-Letellier R, Savoye G, Ghosh S (2016). Fatty acids, eicosanoids and PPAR gamma. Eur J Pharmacol.

[CR68] Matlock JP, Nixon JE, Pawlowski NE (1985). Altered lipid metabolism and impaired clearance of plasma cholesterol in mice fed cyclopropenoid fatty acids. Toxicol Appl Pharmacol.

[CR69] Matthäus B (2012). The new database *Seed Oil Fatty Acids* (SOFA). Lipid Technol.

[CR70] McCammon JA (2005). Target flexibility in molecular recognition. Biochim Biophys Acta Proteins Proteom.

[CR71] Meijsing SH, Pufall MA, So AY (2009). DNA binding site sequence directs glucocorticoid receptor structure and activity. Science (80-).

[CR72] Miehle K, Porrmann J, Mitter D (2016). Novel peroxisome proliferator-activated receptor gamma mutation in a family with familial partial lipodystrophy type 3. Clin Endocrinol (Oxf).

[CR73] Mysinger MM, Carchia M, Irwin JJ, Shoichet BK (2012). Directory of useful decoys, enhanced (DUD-E): better ligands and decoys for better benchmarking. J Med Chem.

[CR74] Mysinger MM, Shoichet BK (2010). Rapid context-dependent ligand desolvation in molecular docking. J Chem Inf Model.

[CR75] Nandakumar M, Tan M-W (2008). Gamma-linolenic and stearidonic acids are required for basal immunity in Caenorhabditis elegans through their effects on p38 MAP kinase activity. PLoS Genet.

[CR76] Nicolotti O, Benfenati E, Carotti A (2014). REACH and in silico methods: an attractive opportunity for medicinal chemists. Drug Discov Today.

[CR77] Nicolotti O, Giangreco I, Miscioscia TF, Carotti A (2009). Improving quantitative structure−activity relationships through multiobjective optimization. J Chem Inf Model.

[CR78] Nicolotti O, Miscioscia TF, Carotti A (2008). An integrated approach to ligand- and structure-based drug design: development and application to a series of serine protease inhibitors. J Chem Inf Model.

[CR79] Nixon JE, Eisele TA, Wales JH, Sinnhuber RO (1974). Effect of subacute toxic levels of dietary cyclopropenoid fatty acids upon membrane function and fatty acid composition in the rat. Lipids.

[CR80] Pal SK, Tew BY, Lim M (2019). Mechanistic Investigation of the androgen receptor DNA-binding domain inhibitor pyrvinium. ACS Omega.

[CR81] Paul Friedman K, Gagne M, Loo L-H (2020). Utility of in vitro bioactivity as a lower bound estimate of in vivo adverse effect levels and in risk-based prioritization. Toxicol Sci.

[CR82] Peláez R, Pariente A, Pérez-Sala Á, Larráyoz IM (2020). Sterculic acid: the mechanisms of action beyond stearoyl-CoA desaturase inhibition and therapeutic opportunities in human diseases. Cells.

[CR83] Protein Preparation Wizard, Schrödinger, LLC, NewYork, NY 2018; Epik, Schrödinger, LLC, New York, NY, 2018; Impact, Schrödinger, LLC, New York, NY, 2018; Prime, Schrödinger, LLC, New York, NY, 2018

[CR84] Reif DM, Martin MT, Tan SW (2010). Endocrine profling and prioritization of environmental chemicals using toxcast data. Environ Health Perspect.

[CR85] Ribeiro MDRCJ, Kushner PhDPJ, Baxter MDJD (1995). The nuclear hormone receptor gene superfamily. Annu Rev Med.

[CR86] Ristow M, Müller-Wieland D, Pfeiffer A (1998). Obesity associated with a mutation in a genetic regulator of adipocyte differentiation. N Engl J Med.

[CR87] Robinson-Rechavi M, Garcia HE, Laudet V (2003). The nuclear receptor superfamily. J Cell Sci.

[CR88] Rotroff DM, Dix DJ, Houck KA (2013). Using in vitro high throughput screening assays to identify potential endocrine-disrupting chemicals. Environ Health Perspect.

[CR89] Sampath H, Ntambi JM (2004). Polyunsaturated fatty acid regulation of gene expression. Nutr Rev.

[CR90] Sanderson JT (2006). The steroid hormone biosynthesis pathway as a target for endocrine-disrupting chemicals. Toxicol Sci.

[CR91] Sarraf P, Mueller E, Smith WM (1999). Loss-of-function mutations in PPARγ associated with human colon cancer. Mol Cell.

[CR92] Sastry M, Lowrie JF, Dixon SL, Sherman W (2010). Large-scale systematic analysis of 2D fingerprint methods and parameters to improve virtual screening enrichments. J Chem Inf Model.

[CR93] Schug TT, Janesick A, Blumberg B, Heindel JJ (2011). Endocrine disrupting chemicals and disease susceptibility. J Steroid Biochem Mol Biol.

[CR94] Shi LM, Fang H, Tong W (2001). QSAR models using a large diverse set of estrogens. J Chem Inf Comput Sci.

[CR95] Shizu R, Min J, Sobhany M (2018). Interaction of the phosphorylated DNA-binding domain in nuclear receptor CAR with its ligand-binding domain regulates CAR activation. J Biol Chem.

[CR96] Shoichet BK (2004). Virtual screening of chemical libraries. Nature.

[CR97] Song Y, Xue X, Wu X (2016). Identification of N -phenyl-2-(N -phenylphenylsulfonamido)acetamides as new RORγ inverse agonists: Virtual screening, structure-based optimization, and biological evaluation. Eur J Med Chem.

[CR98] Soto AM, Sonnenschein C (2010). Environmental causes of cancer: endocrine disruptors as carcinogens. Nat Rev Endocrinol.

[CR99] Spiteller G (2005). Furan fatty acids: occurrence, synthesis, and reactions. Are furan fatty acids responsible for the cardioprotective effects of a fish diet?. Lipids.

[CR100] Spyrakis F, Cavasotto CN (2015). Open challenges in structure-based virtual screening: receptor modeling, target flexibility consideration and active site water molecules description. Arch Biochem Biophys.

[CR101] Su R, Xiong S, Zink D, Loo L-H (2016). High-throughput imaging-based nephrotoxicity prediction for xenobiotics with diverse chemical structures. Arch Toxicol.

[CR102] Toporova L, Balaguer P (2020). Nuclear receptors are the major targets of endocrine disrupting chemicals. Mol Cell Endocrinol.

[CR103] Trisciuzzi D, Alberga D, Mansouri K (2015). Docking-based classification models for exploratory toxicology studies on high-quality estrogenic experimental data. Future Med Chem.

[CR104] Trisciuzzi D, Alberga D, Mansouri K (2017). Predictive structure-based toxicology approaches to assess the androgenic potential of chemicals. J Chem Inf Model.

[CR105] Tyagi S, Gupta P, Saini AS (2011). The peroxisome proliferator-activated receptor: a family of nuclear receptors role in various diseases. J Adv Pharm Technol Res.

[CR106] van der Ven LTM, Rorije E, Sprong RC (2020). A case study with triazole fungicides to explore practical application of next-generation hazard assessment methods for human health. Chem Res Toxicol.

[CR107] Veras Ribeiro Filho H, Tambones IL, Mariano Gonçalves Dias M (2019). Modulation of nuclear receptor function: targeting the protein-DNA interface. Mol Cell Endocrinol.

[CR108] Weatherman RV, Fletterick RJ, Scanlan TS (1999). Nuclear-receptor ligands and ligand-binding domains. Annu Rev Biochem.

[CR109] Weikum ER, Liu X, Ortlund EA (2018). The nuclear receptor superfamily: a structural perspective. Protein Sci.

[CR110] Xu HE, Lambert MH, Montana VG (1999). Molecular recognition of fatty acids by peroxisome proliferator-activated receptors. Mol Cell.

[CR112] Xu L, Sinclair AJ, Faiza M (2017). Furan fatty acids—Beneficial or harmful to health?. Prog Lipid Res.

[CR113] Yu K, Bayona W, Kallen CB (1995). Differential activation of peroxisome proliferator-activated receptors by eicosanoids. J Biol Chem.

