# Supplementary material for: Virtual screening of potentially endocrine-disrupting chemicals against nuclear receptors and its application to identify PPARγ-bound fatty acids
Source: Arch Toxicol. 2020 Sep 9;95(1):355–74. doi: 10.1007/s00204-020-02897-x (PMC7811525; doi:10.1007/s00204-020-02897-x)
Supplement: Supplementary file 1 — (DOCX 3606 kb) [file 204_2020_2897_MOESM1_ESM.docx]

**Virtual screening of potentially endocrine-disrupting chemicals against nuclear receptors and its application to identify PPARγ-bound fatty acids**

Chaitanya K. Jaladanki,^†,§,#^ Yang He,^‡,#^ Li Na Zhao^†^, Sebastian Maurer-Stroh,^†,§^ Lit-Hsin Loo,^†,§^ Haiwei Song,*^,‡^ and Hao Fan*^,†^

^#^Both authors contributed equally to this work.

^†^Bioinformatics Institute (BII), Agency for Science, Technology, and Research (A*STAR), Singapore 138671.

^§^Toxicity Mode-of-Action Discovery (ToxMAD) Platform, Innovations in Food and Chemical Safety Programme, Agency for Science, Technology, and Research (A*STAR), Singapore 138671.

^‡^Institute of Molecular and Cell Biology, 61 Biopolis Drive, Singapore, 138673, Singapore

To whom correspondence should be addressed: Dr. Hao Fan, Bioinformatics Institute (BII), Agency for Science, Technology and Research (A*STAR), 30 Biopolis Street, Matrix No. 07-01, 138671, Singapore; Telephone: +65 64788500; Email: [fanh@bii.a-star.edu.sg](mailto:fanh@bii.a-star.edu.sg) or Dr. Haiwei Song, Institute of Molecular and Cell Biology, 61 Biopolis Drive, Singapore, 138673, Singapore. [haiwei@imcb.a-star.edu.sg](mailto:haiwei@imcb.a-star.edu.sg).

**Supporting Information**

**Table S1**. Fatty acids and their natural resources

**Table S2**. Structural deviations of docking pose from crystal structures of cognate ligands.

**Table S3**. Ranks of ToxCast actives by docking scores and chemical similarity based weighed docking scores in case of progesterone receptor.

**Table S4.** Number of ToxCast actives in top ranked 20 ToxCast chemicals.

**Table S5.**  Ligand enrichment of single structures and consensus over 4 active and 4 inactive structures of PPARγ.

**Table S6.** Novel PPARγ-binding fatty acids and their analogues

**Table S7**. Top ranked 25 fatty acids and their hybrid scores

**Table S8**. Hybrid scores of the 9 tested fatty acids from wild type and two mutant structures of PPARγ

**Figure S1**. Surface plasmon resonance (SPR) assay of specific binding affinities of fatty acids to immobilized PPARγ LBD on the CM5 sensor chip surface, (A) sensorgram overlay and (B) equilibrium binding curve of Docosahexaenoic acid (DHA) to PPARγ LBD, (C) sensorgram overlay and (D) equilibrium binding curve of Docosapentanoic acid (DPA) to PPARγ LBD, (E) sensorgram overlay and (F) equilibrium binding curve of Phytomonic acid (PTA) to PPARγ LBD, (G) sensorgram overlay and (H) equilibrium binding curve of Furanundecanoic acid (FUA) to PPARγ LBD.

| **Table S1**. Fatty acids and their natural resources. | | | |
| --- | --- | --- | --- |
| No. of carbons | Name | Natural resource |  |
| **Saturated fatty acids** | | | |
| C7 | Enthanic acid | Castor oil |  |
| C8 | Caprylic acid | Palm oil, coconut oil |  |
| C9 | Pelargonic acid | Palm oil, coconut oil |  |
| C10 | Capric acid | Palm oil, coconut oil |  |
| C11 | Undecylic acid | Palm oil |  |
| C12 | Lauric acid | Palm kernel oil, coconut oil |  |
| C13 | Tridecylic acid | Palm oil |  |
| C14 | Myristic | Palm kernel oil, coconut oil, butterfat, breast milk |  |
| C15 | Pentdecanoic | Palm kernel oil |  |
| C16 | Palmitic | Palm kernel oil |  |
| C17 | Margaric | Animal or vegetable fat |  |
| C18 | Stearic | Cocoa butter and meat |  |
| C19 | Nonadecylic | Vegetable oils |  |
| C20 | Arachidic | Pea nut butter, cocoa butter, durian, corn oil |  |
| C21 | Heneicosylic | Peanut oil |  |
| C22 | Behenic | Peanut oil, behenic oil, canola oil |  |
| C23 | Tricosylic | Peanut oil |  |
| C24 | Lignoceric | Peanut oil |  |
| C25 | Pentacosylic | Peanut oil |  |
| C26 | hexacosanoic |  |  |
| C27 | heptacosanoic |  |  |
| C28 | octacosanoic |  |  |
| **Monounsaturated fatty acids** | | | |
| C14 | Myristoleic (ω5, 8) | Garfish, wheat, soup, and ginger |  |
| C14 | 8z-tetredecenoicacid (ω6) | Fish |  |
| C16 | Palmitovaccenic (ω5) | Palm oil |  |
| C16 | Palmitoleic (ω9) | Palm oil |  |
| C16 | Sapienic-acid (ω10) | Human sebum |  |
| C16 | 4-hexadeconic (ω12) | Palm oil |  |
| C18 | Vaccenic (ω7) | Milk, butter, and yogurt. |  |
| C18 | Oleic (ω9) | Pecan oil, canola oil, peanut oil, sunflower oil, grape seed oil, sesame oil, durian |  |
| C18 | Elaidic-trans (ω9) | Sunflower oil, |  |
| C18 | Petroselinic(ω12) | Parsley, petroselinium oil |  |
| C18 | 12z-octadecenoicacid(ω6) | Cow milk and goat mil, sunflower oil |  |
| C20 | Paullinic (ω7) | Guarana |  |
| C20 | Gondoic (ω9) | Jojoba oil |  |
| C20 | Gadoleic(ω11) | Fish and cod liver oils |  |
| C20 | 8-ecosenoic(ω12) | Fish oil |  |
| C22 | Erucic (ω9) | Mustard oil |  |
| C24 | Nervonic(ω9) | Salmon sesame seed |  |
| **Polyunsaturated fatty acids** | | | |
| C14 | Myristoleic (ω5, 8) | Garfish, wheat, soup, and ginger |  |
| C16 | Palmitolinolenic acid (ω3, 6, 9) | Palm oil |  |
| C16 | 4,7,10,13-hexadecatetraenoic acid (ω5,8,11,14) | Marine food, fish oil |  |
| C18 | A-linolenic (ω3, 6, 9) | Walnuts, chia seed |  |
| C18 | Stearidonicacid (ω3, 6, 9, 12) | Hemp, blackcurrant, corn gromwell, |  |
| C18 | A-eleostearic (ω5t,7t,9) | Seeds of the makita tree bitter gourd seed, snake gourd seed, pomegranate seed |  |
| C18 | B-eleostearic-trans (ω5t,7t,9t) | Bitter gourd seed, snake gourd seed, pomegranate seed, |  |
| C18 | Punicic (ω5,8t,11) | Snake gourd seed, pomegranate seed, pot marigold seed, catalpa seed |  |
| C18 | 7,10,13-octadecatrienoic (ω5,8,11) | Pomegranate |  |
| C18 | Linoleic (ω6,9) | Peanut, olive chicken |  |
| C18 | Linolelaidic (ω6,9) | Veg oils |  |
| C18 | G-linolenic (ω6,9,12) | Safflower, black currant oils |  |
| C18 | Calendic (ω6t,8t,10t) | Marigold oil |  |
| C18 | Pinolenic (ω6,9,13) | Pines |  |
| C18 | Rumenic (ω7t,9) | Milk, butter, and yogurt. |  |
| C20 | Dihimo-a-linolenicacid (ω3,6,9) | Peanuts |  |
| C20 | 5,8,11,14-eicosotetraenoicacid (ω6,9,12,15) | Green-lipped mussel |  |
| C20 | 9,12,15-eicosatrienoic acid (ω5,8,11) | Vegitable oils |  |
| C20 | B-eicosatetraenoic (ω5,8,11,14) | Green-lipped mussel |  |
| C20 | Dihomo-linoleic(ω6, 9) | Olive oil, animal fats |  |
| C20 | Dihomo-g-linolenic(ω6,9,12) | Animal products |  |
| C20 | Archidonic (ω6,9,12,15) | Peanut butter |  |
| C20 | Eicosapentaenoic acid(ω6,9,12,15,18) (epa) | Fish oils |  |
| C20 | 8,11-eicosadienoic(ω9,12) | Fish oil |  |
| C20 | Mead acid (ω9,12,15) | Cartilage |  |
| C21 | Heneicosapentaenoic acid | Fish Oil |  |
| C22 | Clupinodonic(ω3,6,9,12,15) | Fish oil, salmon, beef |  |
| C22 | Adrenic(ω6,9,12,15) | Algae |  |
| C22 | Osbond acid (ω6,9,12,15,18) | Fish oil, salmon, beef |  |
| C22 | Docohexaenoic acid (ω3,6,9,12,15,18)(dha) | Fish oil, salmon, beef |  |
| C22 | Reslvin D | Fish oil |  |
| C24 | 9,12,15,18,21-tetracosapentaenoic acid (ω3,6,9,12,15) | Fish oil |  |
| C24 | 6,9,12,15,18,21-tetracoshexaenoic acid (ω3,6,9,12,15,18) | Fish oil |  |
| **Oxo and hydroxy fatty acids** | | | |
| C9 | 9-oxo nonanoic acid |  |  |
| C10 | 10-oxo-8-decenoic acid |  |  |
| C12 | Traumatin (12-keto) |  |  |
| C18 | 2-hydroxy oleic acid | Malvales oil |  |
| C18 | 2-hydroxy-octadeca-9,12,15-triene |  |  |
| C18 | 13-keto-9,11-octadeca | Oat oil |  |
| C18 | 18-hydroxy oleic |  |  |
| C18 | Densipolic (12-hydroxy, 9,15-diene) |  |  |
| C18 | 2-hydroxy linoleic | Malvales oil |  |
| C18 | 13-hode (13-hydroxyoctadecadienoic acid) |  |  |
| C18 | Coriolic acid(13-hydroxy 9,11-diene) | Coriaria nepalensis seed oil |  |
| C18 | 13-keto-9-octadecaoinc acid | Monnina emarginata |  |
| C18 | 9-oxo-10,12-octadecadienoic acid | Dimorphotheca seed oils |  |
| C18 | 9-oxo-11,13-octadecadienoic acid | Dimorphotheca seed oils |  |
| C18 | 4-keto-î±-eleostearic acid |  |  |
| C18 | Licanic | Seed oil of licania rigida |  |
| C18 | Dimorphecolic (9-hydroxy, 10,12-diene) | Seed oil of dimorphotheca sinuata |  |
| C18 | 9,10-dihydroxy octdecanoic | Seed oil of cardamine impatiens |  |
| C24 | Nebraskanic (7,18 hydroxy) | Seed oil of the chinese violet cress |  |
| C18 | Phloionolic (9,10,18-hydroxy) | Chamaepeuce afra |  |
| C20 | Auricolic (14-hydroxy) | Oats |  |
| C18 | 9,10,18-trihydroxyoctadecanoic | Chamaepeuce afra |  |
| C20 | 15-oxo-eicosatetraenoic acid | Animal source |  |
| C20 | Lesquerolic (14-hydroxy, 11-ene) | Seed oils from the genus lesquerella |  |
| C18 | Ricinoleic (12-hydroxy) | Castor oil |  |
| C22 | 4-hydroxydha | Fish oil |  |
| C22 | 4-oxodha | Fish oil |  |
| C22 | 4-methoxydha | Fish oil |  |
| C18 | 9-hydroxyode | Fish oil |  |
| C20 | 5-hydroxyepa | Fish oil |  |
| C20 | 5-oxoepa | Fish oil |  |
| C18 | 5-hydroxyote | Fish oil |  |
| C18 | 6-oxoote | Fish oil |  |
| C18 | 2-bromo palmitoleic acid | Fish oil |  |
| C23 | 4-oxotricosahexaenoic acid | Fish oil |  |
| C23 | 4-flourotricosahexaenoic acid | Fish oil |  |
| C22 | 14-hode | Fish oil |  |
| **Cyclic fatty acids** | | | |
| C16 | Hydnocarpic acid acid (c11 ring) | Chaulmoogra oil |  |
| C18 | Chaulmoogric acid (c13 ring) | Chaulmoogra oil |  |
| C18 | Gorlic acid acid (c13 ring) | Gorlic acid |  |
| C10 | 3-carboxy-4-methyl-5-propyl-2-furanpropanoic acid (2,5-epoxy) | Liver fat of fish, in crustaceans and horn corals |  |
| C18 | 9,12-epoxyoctadeca-9,11-dienoic acid | Butter or butter-malt |  |
| C18 | 8-(5-hexylfuran-2-yl)octanoic acid | Butter and butter malt |  |
| C18 | 5-mem-11-15-cyclicdienoic | Fried product of linolenic acid |  |
| C18 | 5-mem-cyclic-linolenic | Fried product of linolenic acid |  |
| C18 | 6-mem-linolenic acid | Fried product of linolenic acid |  |
| C18 | 6-mem -8-en-linoleicacid-2 | Fried product of linolenic acid |  |
|  | majusculoic acid |  |  |
| C17 | 11‑cycloheptylundecanoic |  |  |
|  | 13-Phenyltridecanoic acid |  |  |
|  | 2-hydroxy-11,12-methylene-docos-5-enoate. |  |  |
| **Furan fatty acid** | | | |
| C18 | 9-(5-pentyl-2-furyl)-nonanoate | Liver fat of fish, in crustaceans and horn corals |  |
| C20 | 7-(3,4-dimethyl-5-pentylfuran-2-yl)heptanoic acid (7D5) | Butter or butter-malt |  |
| C20 | 13-(3,4-dimethyl-5-propylfuran-2-yl)tridecanoic acid | Liver fat of fish, in crustaceans and horn corals |  |
| C20 | 9-(3,4-dimethyl-5-pentylfuran-2-yl)-nonanoic acid (9D5) | Fish oil |  |
| C20 | 3,4-dimethyl-5-propyl-2-furanundecanoic acid | Butter or butter-malt |  |
| C20 | 11-(3-methyl-5-propylfuran-2-yl)undecanoic acid | Liver fat of fish, in crustaceans and horn corals |  |
| C20 | 3,4-dimethyl-5-pentyl-2-furanundecanoic acid | Butter or butter-malt |  |
| C19 | 10,13-epoxy-11-methyloctadeca-10,12-dienoic acid (9m5) | Liver fat of fish, in crustaceans and horn corals |  |
| **Acetylinic fatty acid** | | | |
| c18 | Isanic (9,11-diyne) | Isano seed oil |  |
| c18 | Scleropyric (12-yne, 16-ene) | Scleropyrum wallichianum |  |
| c18 | Labellenic (5,6-diene) | Seed oil of leonotis nepetaefolia |  |
| C10 | Dehydromatricaria(2-ene, 4,6,8-triyne) | Solidago species |  |
| C18 | Heisteric (7,11,-diene, 9-yne) | Heisteria silvanii |  |
| C18 | Stearolic acid (9-yne) | Seed oils of picramnia species |  |
| C18 | Oropheic (17-ene, 9,11,13-triyne) | Leaves of orophea enneandr |  |
| C18 | Octadec-11-en-9-ynoic acid | Seed oils of ximenia |  |
| C18 | Ximenynic (11-ene, 9-yne) | Seed oils of ximenia |  |
| C18 | 9,11-octadecadiynoic acid | Isano seed oil |  |
| C18 | Pyrulic (10-ene, 8-yne) | Seed oil (pyrularia pubera |  |
| C18 | Bolekic (13-ene, 9,11-diyne) | Isano seed oil |  |
| C18 | Exocarpic (13-ene, 9,11-diyne) | Isano seed oil |  |
| C18 | 14,15-dehydrocrepenynic acid | Seed oil of crepis foetida |  |
| C18 | (10e,14z)-9-oxooctadeca-10,14-dien-12-ynoic acid | Cantharellus cibarius |  |
| C18 | Tariric acid (6-yn) | Seed oils of picramnia species |  |
| C18 | 8-hydroxy-5,6-octadienoic acid | Seed oil of leonotis nepetaefolia |  |
| C18 | (6z)-6-octadecen-9-ynoic acid | Scleropyrum wallichianum |  |
| C18 | 9z,14z-octadecadien-12-ynoic acid | Seed oil of crepis foetida |  |
| C18 | Crepenynic (9-ene, 12-yne) | Seed oil of crepis foetida |  |
| C18 | Lamenallenic (5,6-diene, 16 ene) | Seed oils of the labiateae |  |
| C18 | Serpentine (aromatic) | Stremyces |  |
| C18 | Catalpic (ω5,7,9) | Seeds of catalpa species |  |
| C18 | Coronaric (9,10-epoxy) | Sunflower oil |  |
| C18 | Colnelenic (9-ether) | Leaves of clematis vitalba |  |
| C18 | Rumenic acid (ω7,9) | Dairy products |  |
| C18 | Taxoleic (ω8,12) |  |  |
| C18 | 9,10-epoxy-stearic | Sunflowe oil |  |
| C18 | Colneleic (8-ether) | Leaves of clematis vitalba |  |
| **Cycloprop fatty acids** | | |  |
| C18 | Malvalic acid (8,9-propene) | Cotton seed oil |  |
| C18 | Lactobacillic acid (11,12-cyclopropyl) | Milk |  |
| C18 | Sterculic (9,10-propene) | Sterculia foetida seed oil |  |
| **Miscellaneous** | | | |
| C14 | 13,13-dimethyltetradecanoic acid | Crab |  |
| C18 | Epithiostearic (5,6 thio) | Canola oil |  |
| C18 | Vernolic (11,12-epoxy) | Vernonia anthelmintica |  |
| C20 | Alchornic (14,15-epoxy) | Vernonia anthelmintica |  |
| C18 | Phytanic (branched) | Fish |  |
| C18 | 10-R-Methyloctadecanoic acid |  |  |
| C19 | Pristanic (branched) | Human mik,bovine fat |  |
| C16 | Rubrenoic (7-phenyl) | Fungal |  |
| C18 | Parinaric (conjugated 9,11,13,15-tetraene) | Seeds of the makita tree |  |
| C20 | 18-methyl-eicosanoic acid |  |  |
| C13 | 4,8,12-Trimethyltridecanoic acid |  |  |
| C15 | 2,6,10,14-tetramethylpentadecanoic (pristanic) acids |  |  |
| C16 | 3,7,11,15-tetramethylhexadec-trans-2-en-1-ol |  |  |
| C16 | 7-methyl-7-hexadecenoic acid | Sun fish |  |
|  | diabolic acids |  |  |
|  | 3-(9h-î²-carbolin-1-yl)propanoic acid |  |  |
|  | 3-(7-hydroxy-9h-î²-carboline-1-yl)propanoic acid |  |  |

| **Table S2**. Structural deviations of docking pose from crystal structures of cognate ligands. | | | | | | | | | | | |
| --- | --- | --- | --- | --- | --- | --- | --- | --- | --- | --- | --- |
| **Protein** | **Active structures** | | | | **Inactive structures** | | | | **Alternative binding site** | | |
|  | **PDB** | **RMSD** | **PDB** | **RMSD** | **PDB** | **RMSD** | **PDB** | **RMSD** | **PDB** | **RMSD** |  |
| AR | 3L3X | 0.8 | 2AX9 | 1.0 | 3RLJ | 0.6 | 2OZ7 | 0.2 | 2PIW | 1.1 |  |
| GR | 3K22 | 0.8 | 6EL9 | 0.4 | 1NHZ | 0.6 | 4MDD | 0.8 | -- | -- |  |
| PR | 1SQN | 0.4 | 3KBA | 0.3 | 2OVH | 0.4 | 4OAR | 0.5 | -- |  |  |
| ERα | 1X7R | 0.9 | 5U2D | 0.7 | 1XP1 | 0.9 | 2IOK | 0.6 | -- | -- |  |
| ERβ | 3OLL | 0.6 | 1U3R | 0.9 | 1L2J | 0.8 | 1NDE | 1.0 | -- | -- |  |
| PPARα | 2P54 | 1.1 | 1I7G | 1.2 | 1KKQ | 1.0 | 2REW | 1.2 | -- | -- |  |
| PPARγ | 3BC5 | 1.2 | 5LSG | 2.1 | 3E00 | 0.9 | 3VSO | 1.0 | -- | -- |  |
| RARα | 3KMR | 0.6 | 3A9E | 0.7 | 1DKY | 0.4 | 5K13 | 0.6 | -- | -- |  |
| RORγ | 3LOL | 0.4 | 4WLB | 0.2 | 5NTK | 1.0 | 5K3N | 0.7 | 5C4T | 0.4 |  |
| VDR | 3B0T | 1.1 | 3AZ2 | 1.6 | 5XPL | 1.8 | 5XUQ | 1.4 | -- | -- |  |
| RXRα | 1MVC | 1.0 | 5LYQ | 0.7 | 3NSQ | 0.8 | 2P1V | 0.6 | -- | -- |  |
| LXRβ | 1P8D | 1.2 | 3KFC | 1.6 | 6S4U | 1.7 | 6S4N | 1.0 |  |  |  |
| RMSD, the RMSD value of the ligand docking pose with the best docking score from crystal structures of cognate ligands. RMSD values are in Angstroms. | | | | | | | | | | | |

| **Table S3**. Ranks of ToxCast actives by docking scores and chemical similarity based weighed docking scores in case of progesterone receptor | | | | |
| --- | --- | --- | --- | --- |
| ToxCast actives Database ID no. | **Docking rank** | | | |
|  | **Active structure (1SQN )** | | **Inactive structure (2OVH)** | |
|  | Rank (docking score) | Rank (hybrid score) | Rank (docking score) | Rank (hybrid score) |
| T00001488 | 1 | 1 | 65 | 68 |
| T00001696 | 2 | 2 | 90 | 91 |
| T00002258 | 3 | 3 | 2 | 1 |
| T00001953 | 4 | 4 | 34 | 11 |
| T00001704 | 6 | 5 | 31 | 10 |
| T00002169 | 8 | 7 | 46 | 14 |
| T00001568 | 10 | 8 | 48 | 52 |
| T00000044 | 13 | 9 | 85 | 19 |
| T00001542 | 14 | 10 | 97 | 98 |
| T00001860 | 20 | 13 | 35 | 12 |
| T00002093 | 23 | 52 | 182 | 182 |
| T00001692 | 39 | 17 | 14 | 6 |
| T00001699 | 41 | 80 | 1941 | 2337 |
| T00001459 | 56 | 98 | 254 | 254 |
| T00002256 | 59 | 103 | 136 | 136 |
| T00001694 | 63 | 19 | 13 | 26 |
| T00001866 | 80 | 56 | 203 | 204 |
| T00001964 | 82 | 22 | 91 | 92 |
| T00000804 | 83 | 132 | 94 | 95 |
| T00001518 | 86 | 134 | 323 | 323 |
| T00001397 | 105 | 67 | 4 | 2 |
| T00000937 | 107 | 161 | 38 | 45 |
| T00001875 | 110 | 25 | 81 | 83 |
| T00001971 | 116 | 27 | 7 | 4 |
| T00001486 | 120 | 28 | 60 | 16 |
| T00002177 | 181 | 110 | 115 | 114 |
| T00002245 | 187 | 262 | 189 | 187 |
| T00002246 | 238 | 317 | 414 | 414 |
| T00001233 | 246 | 331 | 170 | 170 |
| T00000633 | 288 | 160 | 347 | 346 |
| T00001559 | 292 | 49 | 302 | 304 |
| T00000928 | 464 | 559 | 42 | 49 |
| T00001197 | 465 | 562 | 43 | 48 |
| T00001944 | 592 | 685 | 161 | 161 |
| T00001662 | 596 | 682 | 188 | 189 |
| T00001071 | 816 | 875 | 198 | 198 |
| T00001220 | 831 | 889 | 183 | 181 |
| T00002174 | 919 | 965 | 441 | 443 |
| T00001326 | 1041 | 1062 | 315 | 316 |
| T00000984 | 1575 | 1579 | 1935 | 2386 |
| T00001759 | 1767 | 1767 | 1024 | 1024 |
| T00001322 | 1884 | 1884 | 252 | 253 |
| T00001686 | 2188 | 2450 | 41 | 13 |
| T00001538 | 2193 | 2344 | 29 | 9 |
| T00000310 | 2196 | 2330 | 1672 | 2416 |
| T00001432 | 2197 | 2414 | 47 | 53 |
| T00000092 | 2201 | 2015 | 1794 | 1754 |
| T00002227 | 2211 | 2195 | 1731 | 1798 |
| T00002586 | 2224 | 2160 | 1762 | 2387 |
| T00001416 | 2239 | 2255 | 1797 | 2027 |
| T00001941 | 2304 | 2327 | 1936 | 2377 |
| T00002230 | 2313 | 2194 | 1955 | 2230 |
| T00000012 | 2321 | 2201 | 1965 | 2217 |
| T00000968 | 2323 | 2245 | 1993 | 1737 |
| T00002268 | 2342 | 2359 | 24 | 36 |
| T00001134 | 2355 | 2280 | 88 | 88 |
| T00001689 | 2397 | 2294 | 319 | 319 |

| **Table S4.** Number of ToxCast actives in top ranked 20 ToxCast chemicals. | | | |
| --- | --- | --- | --- |
|  | **Number of ToxCast actives in top ranked 20 ToxCast chemicals** | | |
| **Protein** | **Most enriching structure with *E_DOCK_*** | **Total consensus with *E_DOCK_*** | **Total consensus with *E'_DOCK_*** |
| **AR** | 7 | 9 | 9 |
| **GR** | 14 | 14 | 14 |
| **PR** | 10 | 9 | 15 |
| **ERα** | 10 | 10 | 14 |
| **ERβ** | 9 | 9 | 14 |
| **PPARα** | 3 | 4 | 3 |
| **PPARγ** | 4 | 5 | 5 |
| **RARα** | 4 | 4 | 7 |
| **RORγ** | 2 | 2 | 3 |
| **VDR** | 12 | 15 | 15 |
| **RXRα** | 6 | 6 | 4 |
| **LXRβ** | 5 | 5 | 6 |

| **Table S5.**  Ligand enrichment of single structures and consensus over 4 active and 4 inactive structures of PPARγ | | | | | | | |
| --- | --- | --- | --- | --- | --- | --- | --- |
| **Receptor** | **Active structures** | | | **Inactive structures** | | | **Total consensus logAUC (EF1)** |
|  | **PDB ID** | **logAUC (EF1)** | **Concensus logAUC (EF1)** | **PDB ID** | **logAUC (EF1)** | **Consensus logAUC (EF1)** |  |
|  |  |  | *E_DOCK_* |  |  |  |  |
| PPARγ | 3BC5 | 20.9 (2.8) | **25.1** (4.8) | 5DWL | 20.4 (2.8) | 25.9 (4.8) | **27.7 (**5.2) |
|  | 3VSO | 18.2 (3.7) |  | 5LSG | 21.0 (4.6) |  |  |
|  | 3V9V | 22.4 (4.4) |  | 6C5T | 23.3 (4.6) |  |  |
|  | 5GTN | 21.6 (4.2) |  | 6C5Q | 24.7 (4.8) |  |  |
|  |  |  | *E'_DOCK_* |  |  |  |  |
| PPARγ | 3BC5 | 20.0 (2.8) | **25.7** (5.0) | 5DWL | 21.0 (3.7) | 26.3 (**6.2**) | **28.9** (**7.4**) |
|  | 3VSO | 18.3 (3.7) |  | 5LSG | 21.1 (4.6) |  |  |
|  | 3V9V | 23.1 (4.6) |  | 6C5T | 24.5 (4.8) |  |  |
|  | 5GTN | 22.1 (4.4) |  | 6C5Q | 25.4 (5.0) |  |  |
| **Consensus logAUC/EF1**, logAUC/EF1 calculated from consensus docking scores from 4 structures of a single functional state (active or inactive state). **Total consensus logAUC/EF1**, logAUC/EF1 calculated from consensus docking scores from 8 structures (4 active and 4 inactive structures). **Consensus logAUC/EF1** (and **Total consensus logAUC/EF1**) is in bold/italic font when it is 10% larger/smaller than that from the best performing structure, and in normal font in rest cases where it is considered to be comparable. | | | | | | | |

| **Table S6.** Novel PPARγ-binding fatty acids and their analogues | | | | | |
| --- | --- | --- | --- | --- | --- |
| **No. of carbon atoms** | **Fatty acids** | **Hybrid score** | **Kd (μM)** | **Source** | **Chemical similarity** |
| **Furan fatty acids** | | | | | |
| **C19** | **10,13-epoxy-11-methyloctadeca-10,12-dienoic acid (9M5, FNA)** | **-11.19** | 135.7 | **Liver fat of fish, in crustaceans and horn corals** | **1** |
| **C20** | **11-(3-methyl-5-propylfuran-2-yl)undecanoic acid (FUA)** | **-11.16** | 248.7 | **Liver fat of fish, crustaceans and horn corals** | **0.87** |
| C20 | 9-(3,4-dimethyl-5-pentylfuran-2-yl)-nonanoic acid (9D5) | -10.59 |  | Fish oil | 0.95 |
| C20 | 7-(3,4-dimethyl-5-pentylfuran-2-yl)heptanoic acid (7D5) | -9.31 |  | Butter or butter-malt | 0.90 |
| C18 | 8-(5-hexylfuran-2-yl)octanoic acid | -8.36 |  | Butter and butter malt | 0.87 |
| C18 | 9-(5-pentyl-2-furyl)-nonanoate | -8.55 |  | Liver fat of fish, in crustaceans and horn corals | 0.91 |
| C18 | 9,12-epoxyoctadeca-9,11-dienoic acid | -8.34 |  | Butter or butter-malt | 0.90 |
| C20 | 3,4-dimethyl-5-propyl-2-furanundecanoic acid | -11.12 |  | Butter or butter-malt | 0.85 |
| C20 | 3,4-dimethyl-5-pentyl-2-furanundecanoic acid | -10.27 |  | Butter or butter-malt | 0.84 |
| C20 | 13-(3,4-dimethyl-5-propylfuran-2-yl) tridecanoic acid | -10.94 |  | Liver fat of fish, in crustaceans and horn corals | 0.81 |
| C10 | 3-carboxy-4-methyl-5-propyl-2-furanpropanoic acid (2,5-epoxy) | -9.19 |  | Liver fat of fish, in crustaceans and horn corals | 0.77 |
| **Cyclopropane/cyclopropene fatty acids** | | | | | |
| **C18** | **Lactobacillic/Phytomonic acid (11,12-cyclopropyl) (PTA)** | **-9.89** | 102.9 | **Milk** | **1** |
| C18 | Malvalic acid (8,9-propene) | -9.82 |  | Cottonseed oil | 0.94 |
| C18 | Sterculic (9,10-propene) | -8.96 |  | Sterculia foetida seed oil | 0.93 |

| **Table S7.** Top ranked 25 fatty acids and their hybrid scores | | | | |
| --- | --- | --- | --- | --- |
| **No.** | **Fatty acids** | **Hybrid score** | **Category** |  |
| 1 | 6,9,12,15,18,21-tetracoshexaenoic acid (ω3,6,9,12,15,18) | -11.21 | Very long chain fatty acid |  |
| 2 | **10,13-epoxy-11-methyloctadeca-10,12-dienoic acid (9M5, FNA)** | **-11.19** | **Furan fatty acid** |  |
| 3 | **11-(3-methyl-5-propylfuran-2-yl)undecanoic acid (FUA)** | **-11.16** | **Furan fatty acid** |  |
| 4 | **Docosahexaenoic acid (DHA)** | **-11.14** | **PUFA** |  |
| 5 | 9,12,15,18,21-tetracosapentaenoic acid (ω3,6,9,12,15) | -11.13 | Very long chain fatty acid |  |
| 6 | 3,4-dimethyl-5-propyl-2-furanundecanoic acid | -11.12 | Furan fatty acid |  |
| 7 | 13-(3,4-dimethyl-5-propylfuran-2-yl) tridecanoic acid | -10.94 | Furan fatty acid |  |
| 8 | Resolvin D | -10.90 | Very long chain fatty acid |  |
| 9 | 9-(3,4-dimethyl-5-pentylfuran-2-yl)-nonanoic acid (9D5) | -10.59 | Furan fatty acid |  |
| 10 | Clupinodonic acid(ω3,6,9,12,15) | -10.45 | PUFA |  |
| 11 | 4-oxoDHA | -10.39 | Oxo fatty acid |  |
| 12 | 4-flourotricosahexaenoic acid | -10.28 | Oxo fatty acid |  |
| 13 | 3,4-dimethyl-5-pentyl-2-furanundecanoic acid | -10.27 | Furan fatty acid |  |
| 14 | **Eicosapentaenoic acid (EPA)** | **-10.12** | **PUFA** |  |
| 15 | 15-oxo-eicosatetraenoic acid | -10.12 | Oxo fatty acid |  |
| 16 | **Doceapentaenoic acid (DPA)** | **-10.10** | **PUFA** |  |
| 17 | Heneicosapentaenoic acid | -10.02 | PUFA |  |
| 18 | 4-oxotricosahexaenoic acid | -10.01 | Oxo fatty acid |  |
| 19 | **Eicosatrienoic acid (ESA)** | **-10.01** | **PUFA** |  |
| 20 | **Lactobacillic/ phytomonic acid (11,12-cyclopropyl) (PTA)** | **-9.89** | **Cyclopropyl Fatty acid** |  |
| 21 | Malvalic acid (8,9-propene) | -9.82 | Cyclopropyl Fatty acid |  |
| 22 | **Ricinoleic acid (ROA)** | **-9.70** | **Oxo fatty acid** |  |
| 23 | 7-(3,4-dimethyl-5-pentylfuran-2-yl)heptanoic acid (7D5) | -9.31 | Furan fatty acid |  |
| 24 | **Pinolenic acid (PLA)** | **-9.27** | **PUFA** |  |
| 25 | 3-carboxy-4-methyl-5-propyl-2-furanpropanoic acid | -9.19 | Furan fatty acid |  |

| **Table S8.** Hybrid scores of the 9 tested fatty acids from wild type and two mutant structures of PPARγ | | | | |
| --- | --- | --- | --- | --- |
| **No.** | **Fatty acids** | **Wild type** | **Y355A** | **V290M** |
|  | Furannonanoic acid (FNA) | -11.19 | -8.65 | -9.31 |
|  | Furanundcanoic acid (FUA) | -11.16 | -8.50 | -9.22 |
|  | Docosahexaenoic acid (DHA) | -11.14 | -8.41 | -9.21 |
|  | Eicosapentaenoic acid (EPA) | -10.12 | -8.40 | -9.01 |
|  | Doceapentaenoic acid (DPA) | -10.10 | -8.41 | -9.04 |
|  | Eicosatrienoic acid (ESA) | -10.01 | -8.25 | -8.97 |
|  | Phytomonic acid (PTA) | -9.89 | -8.42 | -8.99 |
|  | Ricinoleic acid (ROA) | -9.70 | -8.36 | -8.75 |
|  | Pinolenic acid (PLA) | -9.27 | -8.24 | -8.71 |

**
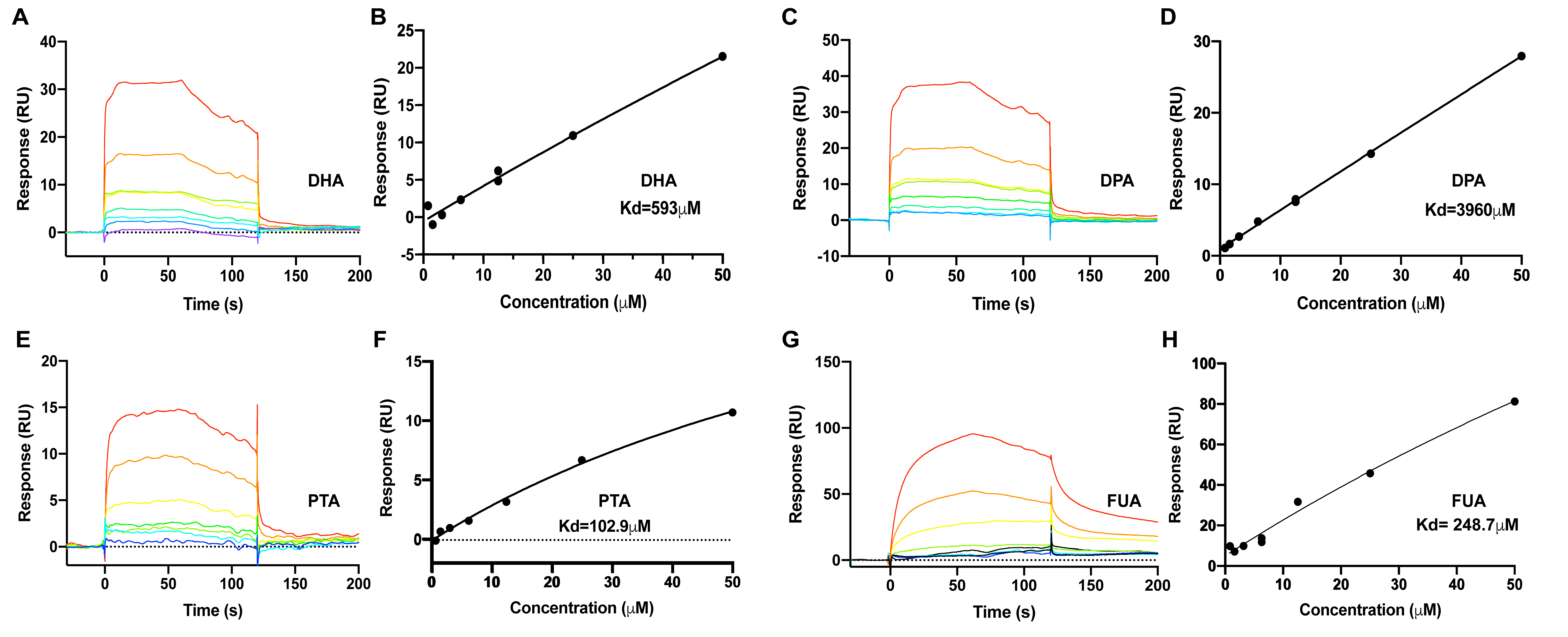
**

**Figure S1**. Surface plasmon resonance (SPR) assay of specific binding affinities of fatty acids to immobilized PPARγ LBD on the CM5 sensor chip surface, (A) sensorgram overlay and (B) equilibrium binding curve of Docosahexaenoic acid (DHA) to PPARγ LBD, (C) sensorgram overlay and (D) equilibrium binding curve of Docosapentanoic acid (DPA) to PPARγ LBD, (E) sensorgram overlay and (F) equilibrium binding curve of Phytomonic acid (PTA) to PPARγ LBD, (G) sensorgram overlay and (H) equilibrium binding curve of Furanundecanoic acid (FUA) to PPARγ LBD.
